# ﻿Reinstatement of *Ticanto* (Leguminosae-Caesalpinioideae) – the final piece in the Caesalpinia group puzzle

**DOI:** 10.3897/phytokeys.205.82300

**Published:** 2022-08-22

**Authors:** Ruth P. Clark, Kai-Wen Jiang, Edeline Gagnon

**Affiliations:** 1 Accelerated Taxonomy Department, Royal Botanic Gardens, Kew, Richmond, Surrey, TW9 3AE, UK Royal Botanic Gardens Richmond United Kingdom; 2 Key Laboratory of Plant Resources Conservation and Sustainable Utilization, South China Botanical Garden, Chinese Academy of Sciences, Guangzhou 510650, China South China Botanical Garden, Chinese Academy of Sciences Guangzhou China; 3 University of Chinese Academy of Sciences, Beijing 100049, China University of Chinese Academy of Sciences Beijing China; 4 Ningbo Botanical Garden, Ningbo 315201, China Ningbo Botanical Garden Ningbo China; 5 Royal Botanic Garden Edinburgh, 20a Inverleith Row, Edinburgh EH3 5LR, UK Royal Botanic Garden Edinburgh, 20a Inverleith Row Edinburgh United Kingdom; 6 Technical University of Munich, Chair of Phytopathology, TUM School of Life Sciences, Emil-Ramman-St. 2, D-85354, Freising, Germany echnical University of Munich, Chair of Phytopathology, TUM School of Life Sciences Freising Germany

**Keywords:** *Biancaea*, *Caesalpiniacrista*, Caesalpinieae, China, Fabaceae, *Guilandinabonduc*, *Mezoneuron*, phylogeny, *Pterolobium*, South-East Asia, winged fruit

## Abstract

A recent molecular phylogenetic analysis of the Caesalpinia group demonstrated that it comprises 26 genera, but the recognition of a putative 27^th^ genus, *Ticanto*, remained in doubt. This study presents a phylogenetic analysis of ITS and five plastid loci revealing a robustly supported monophyletic group representing the Ticanto clade, sister to the morphologically distinct genus *Pterolobium*. Based upon this evidence, along with a morphological evaluation, the genus *Ticanto* is here reinstated. Descriptions are provided for all nine species of *Ticanto*, together with a key to the species, maps, and colour photographs. Nine new combinations are made: *Ticantocaesia* (Hand.-Mazz.) R. Clark & Gagnon, *T.crista* (L.) R. Clark & Gagnon, *T.elliptifolia* (S. J. Li, Z. Y. Chen & D. X. Zhang) R. Clark & Gagnon, *T.magnifoliolata* (Metcalf) R. Clark & Gagnon, *T.rhombifolia* R. Clark & Gagnon, *T.sinensis* (Hemsl.) R. Clark & Gagnon, *T.szechuenensis* (Craib) R. Clark & Gagnon, *T.vernalis* (Champion ex Benth.) R. Clark & Gagnon and *T.yunnanensis* (S. J. Li, D. X. Zhang & Z.Y. Chen) R. Clark & Gagnon. The final major question in the delimitation of segregate genera from within *Caesalpiniasensu lato* and the Caesalpinia group is thus resolved.

## ﻿Introduction

*Caesalpinia* s.l., and the Caesalpinia group more broadly, for a long time defied taxonomic classification, their circumscriptions and generic limits being difficult to define. This was due in part to high levels of morphological homoplasy and the consequent lack of defining characteristic synapomorphies available to delineate segregate genera. *Caesalpinia* s.l. has most often been treated as a single, pantropical genus with up to ca. 150 species encompassing a great diversity of morphological forms, but it has also been considered to comprise numerous smaller genera under as many as 30 generic synonyms (Lewis 1998, 2005). Resolution of generic limits in the Caesalpinia group using molecular phylogenetic data was impeded by a lack of adequate material for molecular sampling and the wide distribution of the taxa. Molecular phylogenetic studies of the group tended to sample from a small range of species to evaluate higher level relationships (Lewis and Schrire 1995; Simpson and Miao 1997; Haston et al. 2005; Bruneau et al. 2008; Manzanilla and Bruneau 2012; Nores et al. 2012) or more densely from selected subgroups (Simpson et al. 2004; Simpson and Ulibarri 2006) until Gagnon et al. (2013) published a densely sampled phylogeny representing 120 species from 18 of the 21 genera, based on a single plastid marker (*rps16*). This study was followed by a comprehensive investigation of the Caesalpinia group by Gagnon et al. (2016) based on one nuclear and five plastid markers which sampled 172 species (equivalent to 84% of taxa in the group at that time), encompassing the morphological diversity of the Caesalpinia group and most of its geographical range, to present a phylogeny that resolved most of the generic limits. The results demonstrated that 26 genera, representing robustly supported clades within the phylogeny, and including nine genera segregated from *Caesalpinia* s.l., should be recognised within the group (Gagnon et al. 2016).

Despite the dense sampling achieved by Gagnon et al. (2016), a lack of available material resulted in uncertain status for a proposed 27^th^ genus, represented in their phylogeny by a single species, *C.crista* L. Nine species distributed primarily in southern China were indicated as potential candidates for inclusion in this putative genus, for which the name *Ticanto* Adans. was identified as the earliest available. The authors lacked the necessary evidence to formally reinstate this genus and highlighted the need for further investigations including thorough molecular sampling.

The difficulties inherent in morphologically defining the elements of the Caesalpinia group are exemplified by *Ticanto*. It lacks obvious diagnostic synapomorphies and was not morphologically characterised by Gagnon et al. (2016) apart from a brief discussion of the presence or absence of a wing on the fruit in comparison with the samaroid winged fruit of the proposed sister genus, *Pterolobium*.

The aim of our study is to test the monophyly of the putative genus *Ticanto* using molecular phylogenetic methods and detailed investigation of morphological characters compared with those of the most closely related genera in the Caesalpinia group, particularly *Pterolobium*, *Mezoneuron* and *Biancaea* (Gagnon et al. 2016). The morphological and molecular phylogenetic analyses presented here support the reinstatement of *Ticanto*, thus resolving the final major question in the reclassification of the complex and taxonomically challenging pantropical *Caesalpinia* s.l. into monophyletic segregate genera. Species descriptions and a key to the species are presented, and new combinations are made for each species.

## ﻿Methods

The species descriptions were developed using herbarium specimens studied at HITBC, IBK, K, and KUN, NPH, and from online specimen images at A, AU, BM, C, CDBI, CSFI, CZH, E, FJSI, GXMG, GXMI, GZAC, GZTM, HGAS, HHBG, IBSC, IMC, IMDY, JIU, L, MO, NAS, NF, NY, P, PE, PEY, SM, SN, SYS, SZG, TAIF, TNM, UC, US, W, WAG, WUK, ZM, via the Chinese Virtual Herbarium (CVH, https://www.cvh.ac.cn/index.php), National Specimen Information Infrastructure (NSII, http://nsii.org.cn/2017/), Plant Photo Bank of China (PPBC, http://ppbc.iplant.cn/), and JSTOR https://plants.jstor.org), in combination with data from protologues and other relevant literature (Hattink 1974; Vidal and Hul Thol 1976; Larsen et al. 1980, 1984; Hou et al. 1996; Chen et al. 2010). The level of detail presented here in the species descriptions varies depending on the availability of material for study.

Due to the relative homogeneity of vegetative and floral characters between *T.crista*, *T.magnifoliolata*, *T.sinensis* and *T.szechuenensis*, the descriptions of these species were generated using a subset of the available specimens consisting of fruiting specimens and selected flowering or sterile specimens that could be confidently identified.

The x-ray images of fruit for study of the venation patterns were taken using a Faxitron MX101 machine with a 4-inch square digital plate.

A representative selection of specimens that were consulted, or for which the identification could be verified via a digital specimen image, contributed the primary data set used to generate the distribution maps. To encompass the full geographical range of the species, additional records were downloaded from the Global Biodiversity Information Facility (GBIF 2021). The GBIF data were cleaned by excluding records not derived from preserved specimens, those that were from capital cities or country centroids, and duplicate specimens or localities. Points that were clearly erroneous (primarily those located in the sea) were either removed or the coordinates were updated following manual georeferencing. Records lacking latitude and longitude coordinates were generally excluded; however, where records existed for areas in which a species was known to occur and for which georeferenced specimens were otherwise unavailable, a few records were manually georeferenced when sufficient locality information was provided.

Tools used for georeferencing were Google Earth Pro, Google Maps (https://www.google.com/maps) and online gazetteers (GEOLocate, https://geo-locate.org/; Falling Rain Global Gazetteer, http://www.fallingrain.com/world/; and Getty Thesaurus of Geographic Names, https://www.getty.edu/research/tools/vocabularies/tgn/). Preliminary mapping of point localities was carried out using GeoCAT (http://geocat.kew.org/editor). The distribution maps were created using ArcMap 10.5 (Redlands 2011). The specimens used to make the maps are listed in Suppl. material 1.

### ﻿Molecular methods

DNA samples were taken from field-collected specimens dried in silica gel or from herbarium specimens. A total of 19 accessions were sequenced, representing six species of *Ticanto*, two of *Pterolobium*, one of *Mezoneuron* and one of *Biancaea* (Table 1).

**Table 1.** Accessions sequenced and used to generate the molecular based phylogeny, with GenBank numbers.

Genus	species	Collector name	Collector number	Country	Herbarium	*ITS*	*trnL-F*	*matK*	*rps16*	*trnDT*
*Biancaea*	*millettii*	Zhi-Ming Zhong	ZZM003	China	IBSC	ON922869	ON932059	-	ON971386	ON971410
*Caesalpinia*	*crista*	Kai-Wen Jiang	KwT033	China	NPH	ON922872	ON932062	ON971417	ON971381	ON971400
*Caesalpinia*	*crista*	Kai-Wen Jiang	TH101	China	NPH	-	ON932064	ON971418	ON971383	ON971407
*Caesalpinia*	*crista*	Zhong-Cheng Liu et al.	LXP-13-23687	China	SYS	ON922873	ON932063	-	-	-
*Caesalpinia*	*crista*	Zhu-Qiu Song	2021057	China	IBSC	ON922871	ON932061	ON971419	ON971396	ON971411
*Caesalpinia*	*magnifoliolata*	Kiyama et al.	1233	China	KUN	ON922868	ON932058	-	ON971387	-
*Caesalpinia*	*sinensis*	Clark	415	China	K, IBK	ON922875	ON932066	ON971423	ON971390	ON971399
*Caesalpinia*	*sinensis*	Clark	429	China	K, IBK	ON922876	ON932067	ON971413	ON971394	ON971405
*Caesalpinia*	*sinensis*	Hang Sun	1672	China	KUN	ON922874	ON932065	ON971415	ON971388	-
*Caesalpinia*	*sinensis*	Yun-Hong Tan	s.n.	China	HITBC	ON922877	ON932068	ON971428	ON971397	-
*Caesalpinia*	aff.szechuenensis	Clark	422	China	K, IBK	ON922870	ON932060	ON971426	ON971392	ON971398
*Caesalpinia*	*vernalis*	Shi-Jin Li	787	China	IBSC	ON922880	ON932071	ON971425	ON971389	ON971412
*Caesalpinia*	*vernalis*	Ya-Min Zhang	YS023	China	NPH	ON922881	ON932072	ON971422	ON971384	ON971408
*Caesalpinia*	*vernalis*	Zhu-Qiu Song	2021061	China	IBSC	ON922879	ON932070	ON971420	ON971382	ON971406
*Caesalpinia*	sp.	Yong-Mei Yi	YYM05	China	NPH	ON922878	ON932069	ON971421	ON971385	ON971409
*Mezoneuron*	*scortechinii*	Wieringa et al.	4195	Australia	WAG	ON922882	ON932073	ON971424	ON971391	ON971401
*Pterolobium*	*punctatum*	Clark	424	China	K	ON922883	ON932074	ON971427	ON971393	ON971404
*Pterolobium*	*stellatum*	MPU	39	South Africa	NGB	ON922884	ON932075	ON971416	-	ON971402
*Pterolobium*	*stellatum*	RBGKewMSB	145895	Kenya	K	ON922885	ON932076	ON971414	ON971395	ON971403

Five genetic markers were amplified: the nuclear internal transcribed spacer (ITS) region of the 18S–5.8S–26S nuclear ribosomal cistron, and four plastid loci, namely *rps16*, the *trnD-trnT* intergenic spacer, the *matK* gene and flanking *3’-trnK* intron, and the *trnL-trnF* intron-spacer region. DNA was extracted from ca. 0.1–0.2 g silica gel-dried leaves or 0.1–0.2 g leaves from herbarium sheets using either: (1) QIAGEN DNeasy Plant Mini Kit, following the manufacturer’s instructions; or (2) 2× CTAB (hexadecyltrimethylammonium bromide) method modified from Doyle and Doyle (1987). DNA was precipitated in 2.5 vol. ethanol or 2/3 vol. isopropanol for dried herbarium specimens and DNA samples were purified on caesium chloride/ethidium bromide gradients (1.55 g/mL) and stored at -20 °C until amplification.

The PCR reactions were carried out in 25 μl volumes, using 2× PCR Premix ‘Dream Taq’ DNA polymerase buffer (4.0 mM MgCl_2_) (Thermo Fisher Scientific), 5×TBT (Samarakoon et al. 2013), and 2 μM of each primer. For samples that did not amplify well initially, the reaction was repeated with 50 μl reaction volume. For ITS only, 2% DMSO (D_2_H_6_OS) was added. For all markers except *matK-trnK*, PCR was initially carried out using standard protocols with a single set of primers. For *matK*-*trnK*, a nested approach was taken, using two sets of primers to amplify shorter regions within the target. Following initially unsuccessful results using standard protocol with a single pair of primers, a nested approach was similarly adopted for *trnL-F*, and *trnD-T*, and for one sample for ITS. The primer pairs used for each marker are listed in Table 2. PCR products were cleaned using a Macherey-Nagel NucleoSpin Purification Kit, following the manufacturer’s instructions.

**Table 2.** Primer pairs used for standard and nested PCR.

		Forward	Reverse	Reference
***ITS***		AB101	AB102	Douzery et al. 1999
	***ITS* nested**	ITS2	ITS3	White et al. 1990
***rps16***		rpsF	rpsR2	Oxelman et al. 1997
***trnD-T***		trnD	trnT	Shaw et al. 2005 ex Demesure et al. 1995
	***trnD-T* nested**	trnD	trnE	Shaw et al. 2005
	***trnD-T* nested**	trnY	trnT	Shaw et al. 2005
***matK-trnK***	**nested**	trnK685F	matKC6-Caesalpinia	Wojciechowski et al. 2004; Gagnon et al. 2016
	**nested**	trnK4La	trnK2R	Wojciechowski et al. 2004
***trnL-F***		trnL	trnF	Taberlet et al. 1991
	***trnL-F* nested**	trnLc	trnLd	Taberlet et al. 1991
	***trnL-F* nested**	trnLe	trnLf	Taberlet et al. 1991

The concentration and quality of DNA in each sample was assessed using a Nanodrop 2000 Spectrophotometer (Thermo Fisher Scientific). Amplification products were sequenced directly with modified dideoxy cycle sequencing with dye terminators (according to the manufacturer’s protocol; Thermo Fisher Scientific). Cycle sequencing reactions were run on an ABI 3730 automated sequencer (according to the manufacturer’s protocols; Thermo Fisher Scientific), using 5× Sequencing Buffer, DMSO, BigDye Premix 3.1, primers diluted 1/10, and 50–300 ng of genomic DNA, depending on quality and concentration. Sequencing was performed with 26 cycles using the standard settings: 0.10 minutes at 96 °C, 0.05 minutes at 50 °C, and 4.00 minutes at 60 °C. Automated sequence output files were edited and assembled using Geneious (version 8.1.9, Biomatters, Auckland, New Zealand).

### ﻿Phylogenetic analyses

Sequences of the same five genetic markers generated as described above (ITS, *rps16*, *trnD-T*, *matK-trnK* and *trnL-F*) from 60 accessions representing 51 Caesalpinia group species and two outgroups were downloaded from GenBank and incorporated into the analysis (Suppl. material 2: Table S2). A sixth genetic marker used in previous studies, *ycf6-psbM* (Gagnon et al. 2016), was also added to our dataset because sequences were available for 45 species covering most of the major groups in the phylogeny (except *Lophocarpinia* and *Stenodrepanum*), including two samples from the putative genus *Ticanto* (*Herendeen 1-V-99-3* and *Wieringa et al. 4199*, both representing *T.crista*).

Sequences were aligned using MUSCLE (Edgar 2004), with subsequent manual adjustments carried out in Geneious. A concatenated matrix of the five plastid loci comprising 7231 bp and a separate matrix of the nuclear ITS locus comprising 940 bp were analysed independently using both Maximum Likelihood (ML) and Bayesian phylogenetic methods.

The ML analyses were implemented using RaxML-HPC2 v. 8.2.10 (Stamatakis 2014) on XSEDE via the CIPRES Science Gateway (Miller et al. 2010). Node support was estimated using the standard nonparametric MLBS procedure, with 100 replicates. Bayesian analyses were carried out using MrBayes 3.2 (Ronquist et al. 2012) via the CIPRES Science Gateway (Miller et al. 2010), with parameters of two parallel runs of four Markov Chain Monte Carlo (MCMC) chains, four swaps per swapping cycle, for 28,000,000 generations, and trees sampled every 1000 generations. The stop criterion, ensuring that convergence of the runs had been achieved, was set to an average standard deviation of split frequencies that dropped to below 0.01. The burn-in fraction was set to 25%.

Following visual comparison of the resulting phylogenies, all sequences were concatenated to create a six-locus matrix (ITS + plastid) of 8171 bp and the combined dataset was analysed using both ML and Bayesian methods as described above. In the preliminary RaxML analyses of this six-locus matrix, each accession was separate in the matrix and represented by a separate terminal in the tree. Where accessions were missing two or more loci, multiple accessions of single species were concatenated for subsequent analyses if they appeared in the same clade in the initial analyses, thus minimising missing data for each species. Accessions were concatenated in this way for six species, and these are highlighted in bold in Suppl. material 2: Table S2.

Results from the phylogenetic trees were visualised using Figtree v1.4.2 (Rambaut 2014), and figures were generated using the packages “Biocmanager”, “treeio”, “ggtree” and “ggplot2” in R, with final edits in Adobe Illustrator. Statistics were obtained using MEGA 11 (Tamura et al. 2021).

## ﻿Results

### ﻿Molecular phylogeny

The concatenated five-locus matrix included 79 accessions (17 newly sequenced, and two of the accessions used by Gagnon et al. 2016 were re-sequenced) representing 60 species (five newly sequenced). In total there were 1207 (=6.8%) parsimony-informative characters in the matrix.

**Figure 1.**
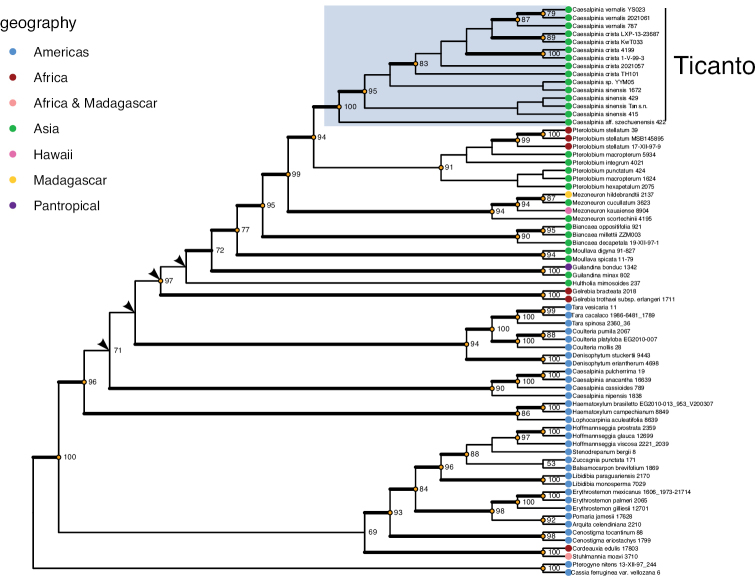
Caesalpinia group ML phylogeny from the combined dataset. Bootstrap values above 50 are shown, values > 75 are indicated with an orange dot at the node. Branches in bold indicate Posterior Probability greater than 0.95 in equivalent BI analysis. Arrows indicate nodes not recovered in BI analysis. The collector number of the corresponding voucher for each terminal is included with the species name. Where a terminal results from analysis of multiple vouchers, the collector numbers are separated by an underscore (see Suppl. material 2).

Separate analyses of the plastid and nuclear datasets revealed the same major clades in both the ML and Bayesian analyses. Incongruences between the nuclear and plastid trees were found at the interspecific level within clades but were unsupported in the nuclear analyses by either bootstrap or posterior probability values; these discrepancies are therefore considered non-contradictory. The three major clades that are of most relevance to this study represent the genera *Ticanto*, *Pterolobium* and *Mezoneuron*, and these were recovered in both the ML and Bayesian analyses (Fig. 1; Suppl. material 3). The recovery of intergeneric relationships is consistent between the ML and Bayesian phylogenies, with two exceptions; in the Bayesian phylogeny, the relative positions of *Gelrebia*, *Hultholia* and the broad clade containing *Ticanto* are unresolved, forming a polytomy, and the position of *Caesalpinia* s.s. is also unresolved, forming a polytomy with the broad *Ticanto* clade and the *Coulteria-Tara-Denisophytum* clade.

Sequences of *Biancaeamillettii* and *Caesalpiniavernalis* are incorporated into our analyses. These two species were initially included in the phylogeny of Gagnon et al. (2016) but were subsequently excluded because they were each represented by only a single sequence. The position of *Biancaeamillettii* is here revealed to form a clade with *B.oppositifolia* and *B.decapetala*, whilst *Caesalpiniavernalis* is resolved as part of the *Ticanto* clade. The species *Pterolobiumpunctatum* is newly sequenced here and is resolved as belonging to a clade with the other sampled species of *Pterolobium*.

The six (including accession *Yi YYM05*, determined as *Ticanto* sp.) sampled species of the proposed genus *Ticanto* are resolved as a monophyletic group in all analyses (Fig. 1; Suppl. materials 3, 4). In the combined dataset analysis the genus is robustly supported as monophyletic and sister (bootstrap = 94%, PP = 1.0) to the genus *Pterolobium*. These genera together comprise a clade that is robustly supported (bootstrap = 99%, PP = 1.0) as sister to *Mezoneuron*. Some resolution of the interspecific relationships within *Ticanto* is achieved; three accessions of *T.sinensis* group together (bootstrap = 95%, PP = 1.0), sister to the clade comprising *T.crista*, *T.vernalis*, and *Yi YYM05* (*Ticanto* sp.), and a further accession of *T.sinensis*. The accession *Clark 422*, determined as Ticantoaff.szechuenensis, is resolved as sister to all other species in the clade (bootstrap = 100%, PP = 1.0). *T.crista* and *T.vernalis* are indicated as probably being sister species, but the relationships between accessions determined as *T.crista* are unresolved.

Partial sequences of ITS, *rps16* and *trnL-F* were obtained from a single accession of *T.magnifoliolata* (*Kiyama et al. 1233*), which in both the ML and Bayesian analyses was resolved as part of the *Ticanto* clade. Because the position of this accession is poorly supported due to a high proportion of missing data (80.8%), the version of the phylogeny including this accession is presented separately (Suppl. material 4).

### ﻿Taxonomic treatment

#### ﻿ Ticanto

Taxon classificationPlantaeFabalesFabaceae

Adans., Fam. Pl. 2: 319. 1763.

3134AF02-2AF2-5954-A972-A24D72070E8F

Caesalpinia sect. Nugaria DC., Prodr. 2: 481, 1825.
Nugaria Prain, J. As. Soc. Beng. 66(ii): 470, 1897 *nom. inval. nom. provis*.

##### Type.

*Guilandinapaniculata* Lam.

##### Etymology and type notes.

Despite reference in the protologue of *Ticanto* to the plate H.M. 6. *t.* 19, this did not constitute typification of the name because Adanson did not mention a previously or simultaneously published species name, nor the type of such a name (Turland et al. 2018; Art. 10.2; https://www.iapt-taxon.org/nomen/pages/main/art_10.html). The rules of the *Code* (Turland et al. 2018) state that a type must therefore be otherwise chosen, which in this case has been achieved in the published card index of *Index Nominum Genericorum* (https://naturalhistory2.si.edu/botany/ing/) by reference to *Guilandinapaniculata* Lam. (1785). The application of the name *Ticanto* is therefore fixed by the type of *G.paniculata* Lam., H.M. 6. *t.* 19, now a heterotypic synonym of *Caesalpiniacrista* L.

The name *Ticanto* was a vernacular name used for these plants by the Brachmanes, also known as Brahmanas, Brahmans, or Brahmins, a sector of Hinduism. This was referenced by Rheede (1686: 33) as “*Ticanto* Brachmanes” and subsequently in the protologue of *Ticanto* (Adanson 1763) as ‘*Ticanto. Bram.*’. The name was without gender. The only combination to have been published in *Ticanto* is *T.nuga* (L.) Medik. (1786), the epithet of which derives from the description of the plants by Rumphius in his Herbarium Amboinense (1747) as “nugae silvarum”, or ‘trifles [i.e., trivial plants] of the woods’. Linnaeus adopted this term in creating the epithet *Guilandinanuga* L. (1762), using it as a noun in apposition. The creation of *Ticantonuga* (L.) Medik. therefore did not assign a gender to the genus name *Ticanto*, and in the absence of other species published under that name it remained ungendered. We hereby assign the feminine gender to the genus name *Ticanto*, thus avoiding changes to the species epithets and maintaining nomenclatural stability.

##### Genus description.

Scandent shrubs or lianas to 15 m. Stems usually with scattered, recurved prickles. ***Leaves*** pari-bipinnate, pinnae 1–16 opposite pairs, leaflets 2–15 opposite pairs, leaf rachis with recurved prickles at base of pinnae and usually scattered in between. ***Stipules*** 0.25–3 mm long. Leaflets elliptic to ovate or obovate, oblong or rhombic. ***Inflorescence*** a terminal or axillary raceme or panicle 7–42 cm long; pedicels articulated; bracts at base of racemes, caducous, bracteoles at base of pedicels, caducous. ***Flowers*** zygomorphic, with a hypanthium, calyx lobes 5, free, the lower lobe cucullate over the others in bud; petals 5, 3.5–12 × 2–7 mm, the median petal distinct from the others in shape, usually with an approximately circular patch of hairs on the inner surface, the lateral petals glabrous or with few hairs; stamens 10, free, 4–14 mm long, the basal half tomentose; ovary 1–2-ovuled, glabrous or hairy; style 4–12 mm long; stigma funnel-shaped and more or less papillate, or truncate. Fruit coriaceous or ligneous, dehiscent or indehiscent, elliptic, lunate, or sub-circular, 1.5–7 × 1.5–5 cm, apex acute or beaked, with or without a stipe, the upper suture with or without a narrow wing 0.5–4 mm wide, or a carinate wing 5–6 mm deep, 1(–2)-seeded.

##### Distribution.

**Map 1.**
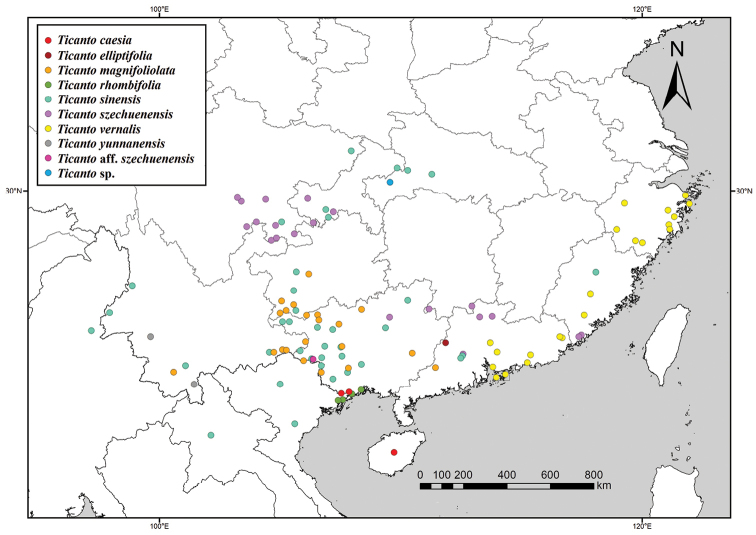
Distribution of all species (excluding *T.crista*).

**Map 2.**
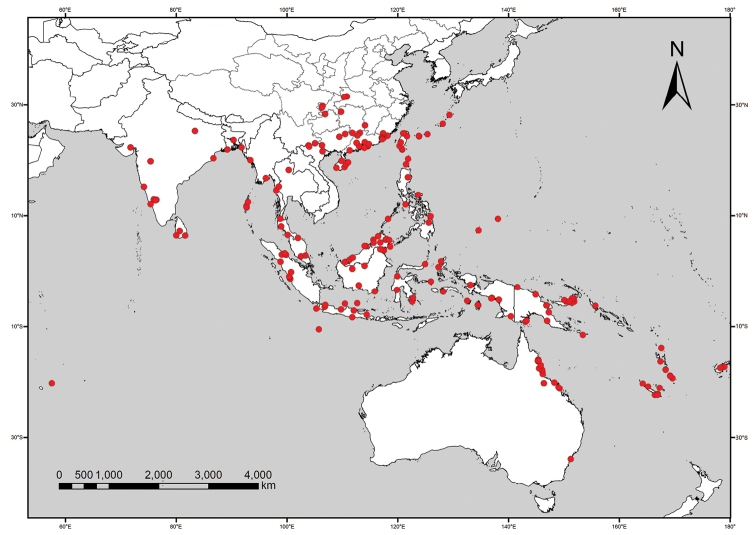
Distribution of *T.crista*.

Andaman Islands, Australia, Cambodia, China (Fujian, Guangdong, Guangxi, Guizhou, Hainan, Hong Kong, Hubei, Hunan, Sichuan, Taiwan, Yunnan, Zhejiang), India, Indonesia, Japan (Ryukyu Islands), Malaysia, Mauritius, Micronesia, Myanmar, New Caledonia, Papua New Guinea, Philippines, Polynesia, Sri Lanka, Thailand, Vietnam (Maps 1, 2).

### ﻿Key to the species

**Table:** 

1	Pinnae 8–16 pairs, fruit dehiscent, ligneous	***T.vernalis***
–	Pinnae 1–8(–9) pairs; fruit usually indehiscent, coriaceous or ligneous	**2**
2	Leaflets 8–15 pairs; 0.8–1.5 × 0.4–0.6 cm	***T.caesia***
–	Leaflets 2–7 pairs; (1.2–)1.5–13(–15) × 0.8–8 cm	**3**
3	Leaflets rhombic, 1.5–2 × 0.8–1.3 cm; fruit without a wing	***T.rhombifolia***
–	Leaflets elliptic, ovate or obovate, 1.2–13(–15) × 0.8–8 cm; fruit with or without a wing	**4**
4	Fruit dehiscent, ligneous; without a wing; fruit venation not prominent	***T.yunnanensis***
–	Fruit indehiscent, coriaceous; with or without a wing; fruit venation prominent	**5**
5	Fruit without a wing, slightly asymmetrical to sub-lunate	**6**
–	Fruit usually with a flat or carinate wing along the upper suture, strongly asymmetrical, sub-circular to lunate or teardrop-shaped	**7**
6	Leaflets 7–13 × 4.5–8 cm, underside of leaflets with brown hairs	***T.elliptifolia***
–	Leaflets 2.1–7.2 × 1–3.3 cm, underside of leaflets usually glabrous or occasionally with sparse ferruginous hairs	***T.crista***
7	Leaflets 3.5–10.8(–15) × 2.1–7 cm, apex usually rounded; ovary glabrous; fruit wing carinate	***T.magnifoliolata***
–	Leaflets 1.2–10.7 × 0.8–5.1 cm, apex usually acute or acuminate; ovary sparsely to densely tomentose, or subglabrous; fruit wing flat or absent	**8**
8	Leaflets 1.2–6 × 0.8–3 cm, leaflet apex usually acute; fruit 1.5–3.4 × 1.5–3 cm, wing 1–3 mm wide, present only along part of the fruit length or absent	***T.szechuenensis***
–	Leaflets 1.8–10.7 × 0.8–5.1 cm, leaflet apex usually acuminate; fruit 3–5.8 × 1.9–4.1 cm, wing 0.5–4 mm wide	***T.sinensis***

### ﻿Species descriptions

#### ﻿1. Ticanto caesia

Taxon classificationPlantaeFabalesFabaceae

(Hand.-Mazz.) R. Clark & Gagnon comb. nov.

D3A24778-B8AE-57E6-908D-8EA4B1EE675E

urn:lsid:ipni.org:names:77303538-1

Caesalpinia hypoglauca Chun & F. C. How., Acta Phytotax. Sin. 7: 20 pl. 6. 1958. Type: China. Kwangtung, Sup Man Ta Shan [Mt. Shiwandashan], 26 Jul. 1933, *H.Y. Liang 69864* (lectotype: (designated by Vidal and Hul Thol 1976): A [A00059892!], isolectotypes IBK [IBK00190838!, IBK00190839!]) (note: the locality of this specimen is in Qinzhou, which has been considered part of Guangxi since 1952).

##### Basionym.

*Caesalpiniacaesia* Hand.-Mazz., Oesterr. Bot. Z. 85: 215. 1936.

##### Type.

China. Kwangsi, *Fenzel 3* (W!).

##### Description.

***Habit*** a climber. ***Stems*** with sparse recurved prickles, puberulent. ***Stipules*** unknown. ***Leaves*** with 5–8(–9) pairs opposite pinnae; leaf rachis and petiole 15–20 cm, leaf rachis and pinnae rachises pilose; leaflets 8–12(–15) opposite pairs per pinna, subsessile, chartaceous, oblong, base strongly asymmetric, apex truncate or obtuse-rounded, emarginate, 0.8–1.5 × 0.4–0.6 cm, both surfaces glabrous. ***Inflorescence*** a panicle, supra-axillary or terminal, 10–15 cm, the axes brown puberulent; pedicels 4–7 mm, articulated. ***Flowers*** with a hypanthium, this glabrous, lower calyx lobe ca. 6 mm long, others 3.5–4 mm, all lobes glabrous; petals obovate-oblong, ca. 3.5–5.5 mm long, median petal with rhombic patch of dense hairs on the inner surface at base of blade, other petals pubescent, shortly clawed; stamen filaments ca. 6 mm long, ferruginous pilose at base; ovary glabrous, 2-ovuled, style ca. 4 mm long. ***Fruit*** blackish when dry, indehiscent, ligneous, elliptic, inflated at maturity, venation prominent, glabrous, ca. 4.5–5 × 2.3–5 cm, ventral suture narrowly winged. ***Seed*** 1, lenticulate, 1.5 × 2.0 cm, blackish.

##### Ecology.

Sparse forests along rivers, elevation 200–1000 m.

##### Phenology.

Flowering July-September, fruiting August.

##### Distribution.

China (Guangxi, Hainan) (Map 1).

##### Notes.

Only one specimen collected from Hainan was seen by the current authors (*H. Fenzel s.n.*, see the citation below), of which the detailed locality is unknown (not recorded on the specimen). To include Hainan in the species distribution, we georeferenced this specimen in the centre of the island.

##### Selected specimens examined.

**China. Guangxi**: Fangcheng, Naliang, *s. coll.*, *s.n.* (PEY). Fangcheng, Dongzhong, Dakeng Village, *Shiwandashan Exped. 3224* (IBK). Shiwandashan, *C.L. Tso 23669* (IBSC). **Hainan**: *H. Fenzel s.n.* (IBSC).

#### ﻿2. Ticanto crista

Taxon classificationPlantaeFabalesFabaceae

(L.) R. Clark & Gagnon comb. nov.

AA97EF86-62D6-583C-9358-54789638136D

urn:lsid:ipni.org:names:77303539-1

= Guilandina nuga L., Sp. Pl., 2. 1: 545. 1762. Type: [Indonesia]. East Indies, Ambon, Nugaesilvarum Rumph. Herb. Am. 5. p.95, t. 50. 1750.
= Guilandina axillaris Lam., Encycl. 1(2): 435. 1785. Type: [India]. Rheede. Hort. Mal. 6: t. 20. 1686.
= Ticanto nuga (L.) Medik., Theodora 52. 1786. Type: based on Guilandinanuga L.
= Guilandina paniculata Lam., Encycl. 1(2): 435. 1785. Type: [India]. Malabar, Kaka Mullu vel Kaka Moullou (in caption Kaka Mullu) Rheede, Hort. Mal. 6: t. 19. 1686.
= Genista scandens Lour., Fl. Cochinch. 2: 428. 1790. Type: Cochinchina (*n.v.*).
= Guilandina parvifolia Stokes, Bot. Mat. Med. 2: 466. 1812. Type: [Indonesia]. East Indies, Ambon, Nugaesilvarum Rumph. Herb. Am. 5. p.95, t.50. 1750.
= Caesalpinia nuga (L.) W.T. Aiton, Hort. Kew, ed 2, 3: 32. 1811. Type: based on Guilandinanuga L.
= Caesalpinia paniculata (Lam.) Roxb., Hort. Beng. 32. 1814. Type: based on Guilandinapaniculata Lam.
= Caesalpinia scandens Heyne ex Roth, Nov. Pl. Sp. 209. 1821. Neotype: (designated by Turner 2021): India, Samalcott, Mar 1811, *B. Heyne s.n.* [K: K000789355!].
= Caesalpinia axillaris (Lam.) DC., Prodr. 2: 481. 1825. Type: based on Guilandinaaxillaris Lam.
= Caesalpinia laevigata Perr., Mém. Soc. Linn. Paris 3: 104. 1825. Type: Philippines. *Perrottet s.n.* (*n.v.*).
= Caesalpinia crista var. parvistipula Urb., Symb. Antill. 2(2): 271. 1900. Type: Trinidad. Cult. Hort. Trinidad, *Broadway 5589* (*n.v.*).

##### Basionym.

*Caesalpiniacrista* L. Sp. Pl. 1: 380. 1753, emend Dandy & Exell in J. Bot. 76: 179. 1938. ≡ *Guilandinacrista* (L.) Small, Fl. S.E. U.S. 591, 1331 (1903).

##### Type.

[Sri Lanka] Ceylon (“Ceylan”), *Herb. Hermann* vol. 1, fol. 68, no. 157 (lectotype (designated by Skeels in Science, n.s., 37: 922. 1913): BM [BM000621459!]) (note: the sheet bearing this specimen was previously identified by a single barcode, BM000594500, which was subsequently replaced with four barcodes representing the four separate specimens on the sheet).

##### Description.

***Habit*** a liana or scrambling shrub to 15 m. ***Stems*** with few, scattered recurved prickles to 5 mm, sometimes with spine-tipped corky tubercles on older stems, or unarmed, glabrous or occasionally sparsely tomentose. ***Stipules*** persistent, triangular, ca. 1 × 1 mm. ***Leaves*** with 3–6(–8) pairs pinnae, these opposite to occasionally slightly subopposite; petiole 1.8–5 cm; rachis 4–31 cm, usually armed with recurved prickles, these sometimes also on pinnae rachises, petiole and rachis usually glabrous, occasionally sparsely to moderately ferruginous tomentose; leaflets 2–4(–7) pairs per pinna, coriaceous, opposite, elliptic, base cuneate to obtuse, apex rounded to obtuse, less commonly acute, obtuse, or acuminate, terminal leaflets 2.1–7.2 × 1–3.3 cm, lateral leaflets 2.1–5.8 × 1–3.1 cm, upper surface glabrous, often glossy, lower surface glabrous or occasionally sparsely ferruginous tomentose, venation reticulate, anastomosing, visible on both surfaces. ***Inflorescence*** a raceme or panicle, axillary or terminal, 8–40 cm, axes glabrous or sparsely tomentose; pedicels 5–15 mm; bracts persistent, triangular or lunate, 1–1.5 × 1 mm; bracteoles caducous, broadly elliptic, apex acute, margins sometimes with small teeth, 1–2.5 × 0.5–1 mm. ***Flowers*** with a hypanthium 1–2 × 3–6 mm, glabrous or sparsely ferruginous tomentose; lower calyx lobe 6–8(–10) × 3–4 mm, other lobes 5–6(– 8) × 2–3 mm, all lobes glabrous, or margins ciliate, or sparsely ferruginous tomentose; median petal 6–9 × 3–7 mm, with dense circular patch of hairs on inner surface at base of blade; upper laterals 6–10 × 3–5 mm, including claw ca. 1 mm, glabrous or inner surface of claw sparsely hairy; lower laterals 7–11 × 3–6 mm, including claw ca. 1 mm, glabrous or inner surface of claw sparsely hairy; stamen filaments 4–12 mm, the vexillary shorter than the lower ones, orange tomentose on lower ½–¾ on inner surface; anthers 1–1.5 mm; ovary 2–4 mm long, glabrous or sparsely or partially tomentose; style 4–11 mm; stigma cupular or funnel-shaped, the rim papillate, sometimes only slightly so, 0.5–1 mm wide. ***Fruit*** indehiscent, coriaceous, elliptic to lunate, subsymmetrical to somewhat asymmetrical, slightly inflated, base cuneate, stipe 2–5 mm, apex acute to beaked, beak 1–10 mm, venation reticulate, prominent, glabrous or very sparsely tomentose, (2–)2.7–7 × 2.2–3.7 × 0.3–0.8 cm, lacking a wing. ***Seeds*** 1, rarely 2, circular to reniform in outline, flat, ca. 2–2.5 × 1.5–2 × 0.5–1 cm. (Fig. 3A).

##### Ecology.

Riverbanks, sandy beaches, in and behind sandy parts of mangroves, on chalk rocks and limestone, at low altitude, elevation rarely up to 350 m.

##### Phenology.

The flowering and fruiting time of this species varies throughout its distribution and may be correlated with latitude or biome as indicated below; however, the periodicity listed below derives in some cases from few records and thus may be incomplete.

China, Japan: Flowering February-April, fruiting April-October;

Bangladesh, India, Myanmar: Flowering August-December, fruiting December-October;

Thailand, Vietnam: Flowering January-June, fruiting January-June;

Malaysia, Indonesia, Palau Islands: Flowering February-December, fruiting February-January;

Philippines: Flowering December-March, fruiting May-December;

New Guinea: Flowering January-November, fruiting February-November;

New Hebrides, Solomon Islands: Flowering February-December, fruiting February-December;

New Caledonia: Flowering May, fruiting unknown;

Mauritius: Flowering unknown, fruiting February.

##### Distribution.

Andaman Islands, Australia, Cambodia, China (Fujian, Guangdong, Guangxi, Guizhou, Hainan, Hong Kong, Hubei, Hunan, Sichuan, Taiwan, Yunnan), India, Indonesia, Japan (Ryukyu Islands), Malaysia, Mauritius, Micronesia, Myanmar, New Caledonia, Papua New Guinea, Philippines, Solomon Islands, Sri Lanka, Thailand, Vanuatu, Vietnam (Map 2).

##### Notes.

The current authors have not seen any specimens or specimen records from Cambodia (other than a single record in GBIF which is not from a preserved specimen), but Vidal and Hul Thol (1976) list Cambodia within the range for this species, citing two specimens from Kampot Province (*Geoffray 62*, *62 bis*) and that information is retained here. As these specimens could not be traced by the current authors, they are not included on the map. See also Nomenclatural notes on Caesalpinia (Ticanto) crista, below.

##### Selected specimens examined.

**Australia. Queensland**: Daintree area, Noah Creek, Mangroves, *J.J. Wieringa 4199* (WAG). **Bangladesh.** Chittagong, *Flagstaff 255* (K). **China. Chongqing**: Jiulongpo, Jinfeng, Baihe Village, *Jiulongpo Exped. 500107150402-289LY* (IMC). Mt. Jinyunshan, *Z.Y. Liu 182996* (IMC). **Fujian**: Hua’an, Wenhua, *W.D. Han 20667* (NF). Pinghe, Daxi, Jiangzhai Village, *H.B. Chen s.n.* (FJSI). Xiamen, Yunding Cliff, *G.D. Ye 1208* (IBSC). Yunxiao, Huotian, Baihuayang Reservoir, *G.D. Ye 2482* (FJSI). Zhangzhou, Zhaoan, Wushan, *X.F. Zeng ZXF19839* (CZH). Zhangzhou, Zhaoan, Wushan, Jinshui Village, *X.F. Zeng ZXF41029* (CZH). Zhao’an, Jinshui Village, *s. coll.*, *s.n.* (AU). **Guangdong.** Boluo, Mt. Luofushan, near Damiao, *Yue78 5714* (IBSC). Dianbai, Luokeng, Mt. Shuangjiling, *H.G. Ye 6379* (IBSC). Huidong, Pingshan Forest Farm, Mt. Chenshuishan, Zhulian?, *P.Y. Chen*, *B.H. Chen & G.C. Zhang 46* (IBSC). Jiangmen, Mt. Guifengshan, *J.Y. Chen 20165220* (SN). Qingyuan, Yangshan, near Qincaitang Reservoir, *K.W. Jiang KwT033* (NPH). Ruyuan, Daqiao Health Center, back mountain, *Yue73 1182* (CSFI). Shenzhen, Longgang, Nan’ao, Yangmeikeng, *S.Z. Zhang*, *L.Q. Li et al. 185* (SZG). Xin’an, Ng-tung Shan, *T.M. Tsui 231* (NAS). Zhaoqing, Mt. Dinghushan, *S.J. Li 30* (IBSC). *ibid.*, *Z.Q. Song 2021057* (IBSC). **Guangxi**: Liuzhou, Longtan Park, Mt. Jiaodingshan, *Longtan & Dule Exped. 242* (IBK). Yang-shoh, *H. Fung 21112* (SYS). **Guizhou**: Tungtze, *Y. Tsiang 4894* (IBSC). **Hainan**: Kan-en, Chim Fung Ling, near Sam Mo Watt Village, *S.K. Lau 3582* (IBSC). Lin’gao, Maniao, Wende Village, *Z.X. Li et al. 911* (IBSC). Qionghai, Lehui, near Shuangbang Village, *Y. Zhong 4472* (IBSC). Wanning, Mt. Dongshan, the second mountain range, *S.P. Kao 52115* (IBSC). Wenchang, Longlou, nera Mt. Beijianshan, *G.W. Tang*, *Z.M. Li & J. Li TangGW2525* (IBSC). **Hubei**: Shennongjia, Xingshan to Yangri, *D.G. Zhang ZB130226624* (JIU). Xingshan, Xiakou, Jianyangping, Lifangyan to Huangliang, *D.G. Zhang zdg4185* (JIU). **Hunan**: Jianghua, *C.J. Qi 3822* (CSFI, IBSC). Xiangxi, Yongshun, Zejia, Donglu Village, *K.D. Lei ZZ40516121* (JIU). **Jiangxi**: Ji’an, Suichuan, Daijiapu, Xianmo, *Z.C. Liu*, *W.J. Xiong*, *F. Ye*, *L. Deng*, *M. Tu*, *X.J. Zhang*, *L. Feng*, *Q.Y. Yin & N.N. Liu LXP-13-23687* (SYS). **Taiwan**: Hsinchu, Hsienchiaoshih, *Z.-H. Chen 277* (TAIF). **Yunnan**: Hekou, Erqu, *W.X. Liu 277* (HITBC). **Micronesia.** Caroline Islands, Yap Group, Gorror Island, Central Plateau, *E.Y. Hosaka 3319* (K). **India.** Kaswar, *R.J. Bell 7750* (K). Kuppam River, Taliparamba, *C.A. Barber 8788* (K). North Kanara, *W.A. Talbot 1256* (K). S. Andaman, *Dr King’s Collector s.n.* (K). **Myanmar.** Myebon, *H.S. McKee 6069* (K). Rangoon, *D.R. Khant 1079* (K). **Mauritius.** The Pouce, *J. Gueko s.n.* (K). **New Caledonia.** Yate, Touaourou, *s. coll. s.n.* (K). **Thailand.** Narithiwat: Kulok river mouth next to bridge on road from Tak Bai to Sungai Ko-lok, *P.S. Herendeen & R. Pooma 1-V-1999-3* (US). **Vanuatu.** Aniwa Island, Isavai village, *P. Curry 1447* (K). Banks Islands, Port Patterson, *A. Morrison s.n.* (K). New Hebrides, Erromanga, between Nouanko Camp and Ipota, about 10 km E of Ipota, *P.S. Green RSNH1318* (K). New Hebrides, Port Vila, *A. Morrison s.n.* (K).

#### ﻿3. Ticanto elliptifolia

Taxon classificationPlantaeFabalesFabaceae

(S. J. Li, Z. Y. Chen & D. X. Zhang) R. Clark & Gagnon comb. nov.

EABB12B5-F7B9-505E-A779-A2DF9C64D610

urn:lsid:ipni.org:names:77303540-1

##### Basionym.

*Caesalpiniaelliptifolia* S. J. Li, Z. Y. Chen & D. X. Zhang, Nordic J. Bot. 22: 349. 2003.

##### Type.

China Guangdong, Fengkai, Qixing, alt. 120m, 20 July 2000, *Shijin Li 026* (holotype: IBSC!)

##### Description.

***Habit*** a liana to 15 m. ***Stems*** occasionally with scattered, recurved prickles to 2 mm. ***Stipules*** caducous. ***Leaves*** with 1–2 pairs opposite pinnae; leaf rachis 20–30 cm, leaf rachis and pinnae rachises with recurved prickles; petiolules 2–3 mm; leaflets 2 opposite pairs per pinna, coriaceous, broadly elliptic, base cuneate to rounded, apex rounded, obtuse or acute, 7–13 × 4.5–8 cm, upper surface glabrous, glossy, lower surface with brown hairs especially on midvein; venation anastomosing, finely reticulate. ***Inflorescence*** a panicle, supra-axillary or terminal, 15–25 cm, all parts densely hairy; pedicels 8–12 mm, articulated; bracts caducous, lanceolate, 1–3 mm; bracteoles caducous, ca. 1.5 mm. ***Flowers*** with a hypanthium, this with brown hairs; calyx lobes ca. 6 × 2 mm, with brown hairs; median petal blade reflexed, claw ca. 3.5 × 1 mm, blade ca. 7 × 6–7 mm, circular patch of brown hairs at base of blade, otherwise glabrous; lateral petals 10–12 × 4–5 mm, claw ca. 1 mm, glabrous; stamen filaments 9–14 mm, the basal ca. ½ tomentose, anthers 2 mm; ovary subsessile, ca. 2 mm long, tomentose, 1- or 2-ovuled; style (2–)7–10 mm, occasionally as short as 2 mm, glabrous; stigma truncate, papillate. ***Fruit*** indehiscent, coriaceous, oblong-elliptic to sub-lunate, sub-symmetrical, compressed but slightly inflated when mature, base cuneate, stipe short, apex acute to attenuate, beak ca. 1–5 mm, veins prominent and reticulate, ca. 4.5–5 × 2.2–2.5 cm, lacking a wing. ***Seeds*** 1 or 2, brownish black, compressed, sub-circular, ca. 10–15 mm cm in diameter.

##### Ecology.

Beside ditches, elevation ca. 100 m.

##### Phenology.

Flowering April, fruiting May-June.

##### Distribution.

China (Guangdong) (Map 1).

#### ﻿4. Ticanto magnifoliolata

Taxon classificationPlantaeFabalesFabaceae

(Metcalf) R. Clark & Gagnon comb. nov.

F300E173-15C3-5E0A-810A-A771A55A1404

urn:lsid:ipni.org:names:77303541-1

##### Basionym.

*Caesalpiniamagnifoliolata* Metcalf. Lingnan Sci. J. 19: 553. 1940.

##### Type.

China. Kwangsi, Ling Yun Hsien, *Steward*, *A.N. & Cheo, H.C. 583* (holotype: A [A00059894!]).

##### Description.

**Figure 2.**
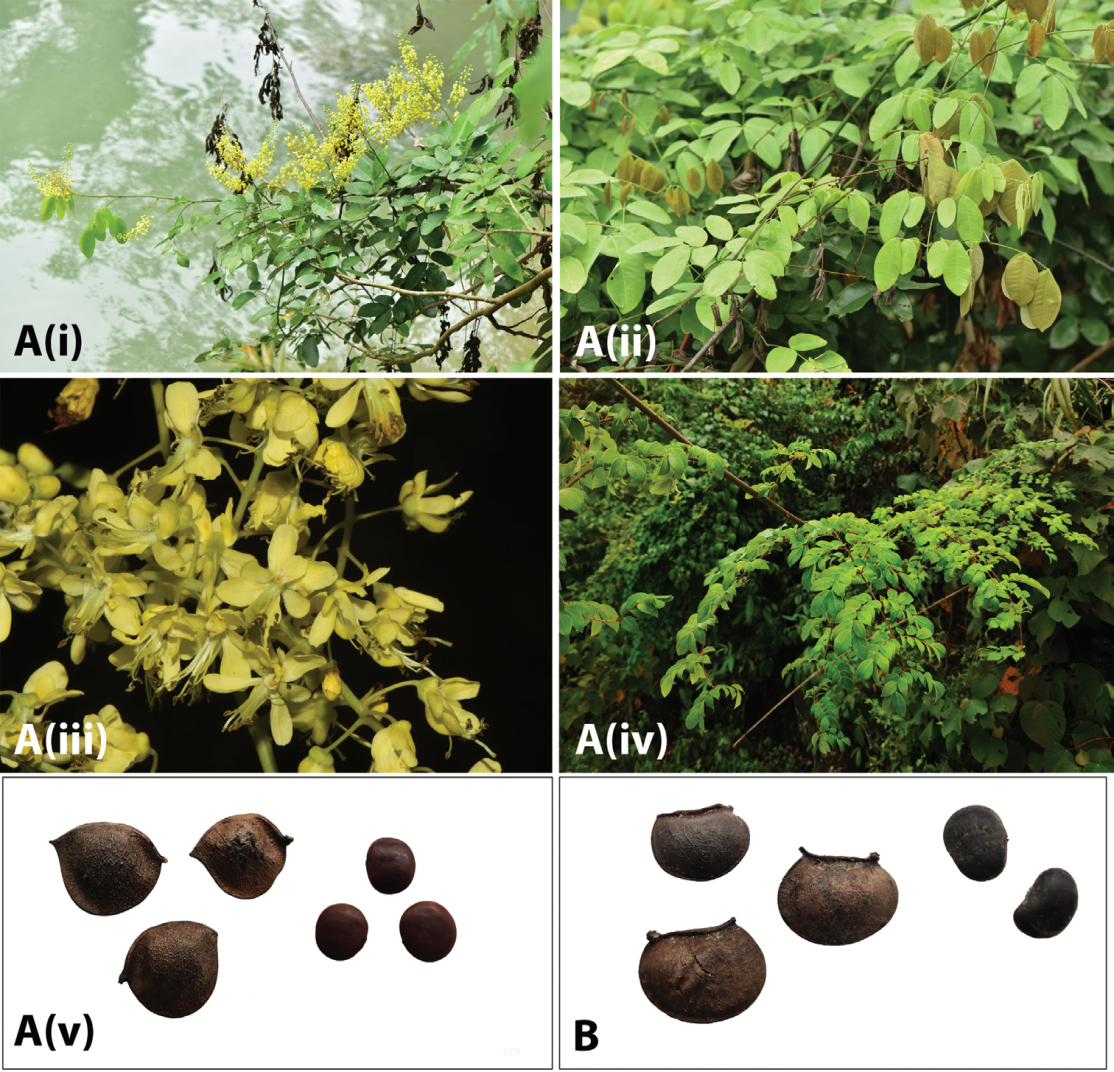
Photos of *Ticanto***A***T.crista* (i) habit (ii) leaves (iii) flowers (*E.D. Liu 8629*, Yunnan, China (KUN)) (iv) leaves (*K.W. Jiang KwT033* (NPH)) (v) fruit and seeds (*Z.Q. Song s.n.* (fruit and seeds in NPH)) **B***T.magnifoliolata* fruit and seeds (*Q. Meng s.n.*, Guizhou, China (fruit and seeds in NPH)) .

***Habit*** a scrambling shrub. ***Stems*** with scattered recurved prickles, ferruginous puberulent, glabrescent. ***Stipules*** not seen. ***Leaves*** with 2–3(–4) pairs opposite pinnae; petiole 3.5–9 cm; leaf rachis 3.3–18.5 cm, with paired recurved prickles at the pinna insertion points and scattered in between, or unarmed; pinnae 2–9 cm; leaflets 2(–3) opposite pairs per pinna, coriaceous, elliptic to obovate, base oblique, apex usually rounded or obtuse, retuse to emarginate, occasionally acute; terminal leaflets 3.5–10.8(–15) × 2.1–7 cm; lateral leaflets 3.5–9.3 × 2.1–4.6 cm; both leaf surfaces glabrous, or lower surface sparsely puberulent; venation reticulate, anastomosing. ***Inflorescence*** a raceme or panicle, axillary or terminal, 15–30 cm; axes and pedicels sparsely to moderately ferruginous tomentose; pedicels 5–11 mm, articulated, glabrous; bracts and bracteoles not seen. ***Flowers*** with a hypanthium ca. 1 × 2–4 mm, glabrous to sparsely orange tomentose; lower calyx lobe ca. 7 × 3 mm; other calyx lobes ca. 5–6 × 2 mm; all calyx lobes with ciliate margins; median petal inrolled, with a patch of hairs at base of blade on inner surface, ca. 7–10 × 3–5 mm; upper laterals ca. 7–10 × 3–5 mm, hairy on the claw inner surface; lower laterals ca. 7–10 × 3–5 mm, hairy on the claw inner surface. Stamen filaments ca. 5–9(–10) mm, the basal ½ tomentose; ovary ca. 3 mm long, glabrous, subsessile; style 5–10 mm, glabrous; stigma funnel-shaped, papillate, sometimes laterally placed. ***Fruit*** dark brown, indehiscent, coriaceous, lunate, stipe ca. 1 mm, beak 2–7 mm, venation prominent, glabrous, 2.8–4.2 × 2.2–3.1(–3.5) × 0.4–0.7 cm, wing on ventral suture carinate, 5–6 mm deep. ***Seed*** 1, brownish black, compressed, sub-circular, ca. 2 × 2.5 cm (Fig. 2B).

##### Ecology.

Forests, scrubland; 400–1800 m.

##### Phenology.

Flowering February-July, fruiting May-November.

##### Distribution.

China (Guangdong, Guangxi, Guizhou, Yunnan) (Map 1).

##### Selected specimens examined.

**China. Guangdong**: Luoding, Caotang, Lianshi Village, *B.H. Chen et al. 1793* (IBSC). **Guangxi**: Donglan, Sannong, Xiangtiandong Village, Haoyantun, *H.Z. Lü*, *Z.Z. Lan & H.F. Cen 451224180425005LY* (GXMG). Fusui, Zhongdong, Luhan?, near Mt. Damingshan, *S.H. Chun 12435* (IBSC). Huanjiang, Mulun Nature Reserve, Xiazhai Observation Deck, *W.B. Xu*, *R.C. Hu & M.Q. Han ML1296* (IBK). Leye, on the way from Gantian to Daping, *M. Shi s.n.* (NPH). Lingle, Xinhua, Sanhe, *Lingle Exped. 34420* (IBK). Longzhou, Jinlong, Jinmei, Nongqiao, *W.B. Xu*, *Y.S. Huang et al. LZ1370* (IBK). Napo, Bing Mung, Rong La Shan, *H. Akiyama*, *H. Kudo*, *J. Murata*, *T. Sugawara*, *N. Tanaka*, *Y. Tateishi*, *Y.G. Wei & S.G. Wu 1233* (KUN). Pingguo, Taiping, Chami Village, Chajiangtun, *H.Z. Lü*, *Y. Lin*, *L.L. Mo & B.Z. Ban 451023150325048LY* (GXMG). Pingnan, Yaoshan, Luoxiang, Mt. Lingdingshan, *C. Wang 39106* (IBK). **Guizhou**: Anlong, Huali, Dewo, *Guizhou Exped.* (*C.S. Chang & Y.T. Chang*) *3543* (HGAS). Anlong, Longshan, Dushan, *Guizhou Exped.* (*C.S. Chang & Y.T. Chang*) *3138* (HGAS, IBSC, KUN, NAS, PE). Ceheng, Shuanghe, Jishanlin, *C.Z. Dang 1684* (HGAS). Pingba, Qibo, Taohua Village, Baidong, *R. Yang & L.B. Yan PB1356* (GZAC). Xingren, Nongchang, near Shanhe, *Guizhou Exped.* (*C.S. Chang & Y.T. Chang*) *7790* (HGAS, IBSC, KUN, NAS, PE, WUK). Xingyi, Qingnan, Yangping, Bajiaoping?, *Anshun Exped. 744* (HGAS). **Yunnan**: Funing, Zhesang, Nonguo Village, *J. Cai*, *J.D. Ya*, *X.Q. Yu*, *Y. Su & C.H. Li 14CS9136* (KUN). Mali, Xialiangshuijing, *Malipo Exped. 5326240386* (IMDY). Malipo, on the way from Huilong to Mabiao, *X.X. Zhu*, *B. Xiao*, *G.S. Wang & J. Wang LiuED8612* (KUN). Si-chour-hsien, Ping-chai, *K.M. Feng 12522* (KUN). Simao, Simaogang, *H. Wang 3842* (HITBC).

#### ﻿5. Ticanto rhombifolia

Taxon classificationPlantaeFabalesFabaceae

(J. E. Vidal) R. Clark & Gagnon comb. nov.

3237DD33-E57A-59CB-A598-4FAC9FA03943

urn:lsid:ipni.org:names:77303542-1

##### Basionym.

*Caesalpiniarhombifolia* J. E. Vidal, Adansonia, n.s., 15: 394. 1976.

##### Type.

Vietnam. Quang Ninh, Dam Ha, *W.T. Tsang 29830* (holotype: P [P02142684!]; isotypes: C [C10011919!], E [E00313521!], G, K [K000789332!], L [L0018793!], SING).

##### Description.

***Habit*** a liana. ***Stems*** with sparse recurved prickles, glabrous. ***Stipules*** caducous, not seen. ***Leaves*** with 3–6 pairs opposite pinnae; petiole 2.5–3 cm; leaf rachis ca. 10–15 cm, with paired recurved prickles at base of pinnae and scattered in between, glabrous; pinna rachis 3.5–6 cm, glabrous; petiolules ca. 1 mm; leaflets 3–5 opposite pairs per pinna; chartaceous, slightly rhombic, base broadly cuneate, apex acute, rarely slightly emarginate, 1.5–2 × 0.8–1.3 cm, both surfaces glabrous; venation reticulate, anastomosing. ***Inflorescence*** a panicle, axillary or terminal, 10–20 cm; axes glabrous; pedicels 6–9 mm, articulated, glabrous or subglabrous. ***Flowers*** with a hypanthium that is sparsely ferruginous tomentose or glabrous; calyx lobes with ciliate margins; petals ca. 7 mm long, median petal smaller than the others, rounded at apex, with a patch of hairs at base of blade, other petals hairy on inner surface of claw; stamen filaments ca. 7 mm long, pale orange tomentose on basal ca. ½ on inner surface; anthers ca. 1.5 mm long; ovary glabrous, 1- or 2-ovuled; style ca. 10 mm, glabrous. ***Fruit*** indehiscent, coriaceous, asymmetrical, sub-lunate, inflated when mature, stipe ca. 3 mm, apex slightly beaked, venation prominent, glabrous, ca. 3–4 × 2.3–2.5 cm, lacking a wing. ***Seed*** 1, broadly ellipsoid, ca. 1.8–2 × 1–1.5 × 1.1 cm, matt black.

##### Ecology.

Thickets.

##### Phenology.

Flowering May-July, fruiting October-November.

##### Distribution.

China (Guangxi), Vietnam (Map 1).

##### Selected specimens examined.

**China. Guangxi**: Dongxing, Jiangping, Hezhou, *Dongxing Exped. 450681180510051LY* (IBK). Fangcheng, Dawangjiang Village, *Y.S. Huang & L. Wu H110397* (IBK).

#### ﻿6. Ticanto sinensis

Taxon classificationPlantaeFabalesFabaceae

(Hemsl.) R. Clark & Gagnon comb. nov.

7DB92BA1-6B22-541E-AFB3-BC5EC070E3A7

urn:lsid:ipni.org:names:77303545-1

= Caesalpinia chinensis Roxb. in Fl. Ind. ed. 2: 361. 1832., *nom. rej.* Li et al. Taxon 51: 816. 2002. Type: not designated.
= Mezoneuron sinense var. parvifolium Hemsl., J. Linn. Soc., Bot. 23: 205. 1887. Type: China. Hupeh Province, Ichang, *Henry*, *A. 2238* (lectotype, designated here: K [K000264687!]; isolectotype: P [P00751902!])
= Caesalpinia tsoongii Merr., Philipp. J. Sci. 27: 162. 1925. Type: China. Szechuen, *Tsoong 4190*. (holotype: UC [UC227358!]; isotype: GH [A00059897!]).
= Caesalpinia stenoptera Merr., J. Arnold Arbor. 19: 35. 1938. Type: Indo-China. Tonkin, Cao Bang, Ban Gioc, Jun. 1933, Petelot, *A. 4757* (lectotype, designated here: A [A00059899!], isolectotypes: P [P02142685!, P02142686!], NY [NY00003575!, NY00003576!, NY00003577!]).

##### Basionym.

*Mezoneuronsinense* Hemsl., J. Linn. Soc., Bot. 23: 204. 1887. ≡ *Caesalpiniasinensis* (Hemsl.) J.E. Vidal in J.E. Vidal & S. Hul Thol, Bull. Mus. Natl. Hist. Nat., ser. 3, 395 (Bot. 27): 90. 1976. *nom. cons.* Li et al. Taxon 51: 816. 2002.

##### Type.

China. Hupeh, Ichang, *A. Henry*, (Herb. Kew) (lectotype (designated by Larsen et al. 1980): China, *Henry*, *A. 3113* [K 000264688!]).

##### Description.

**Figure 3.**
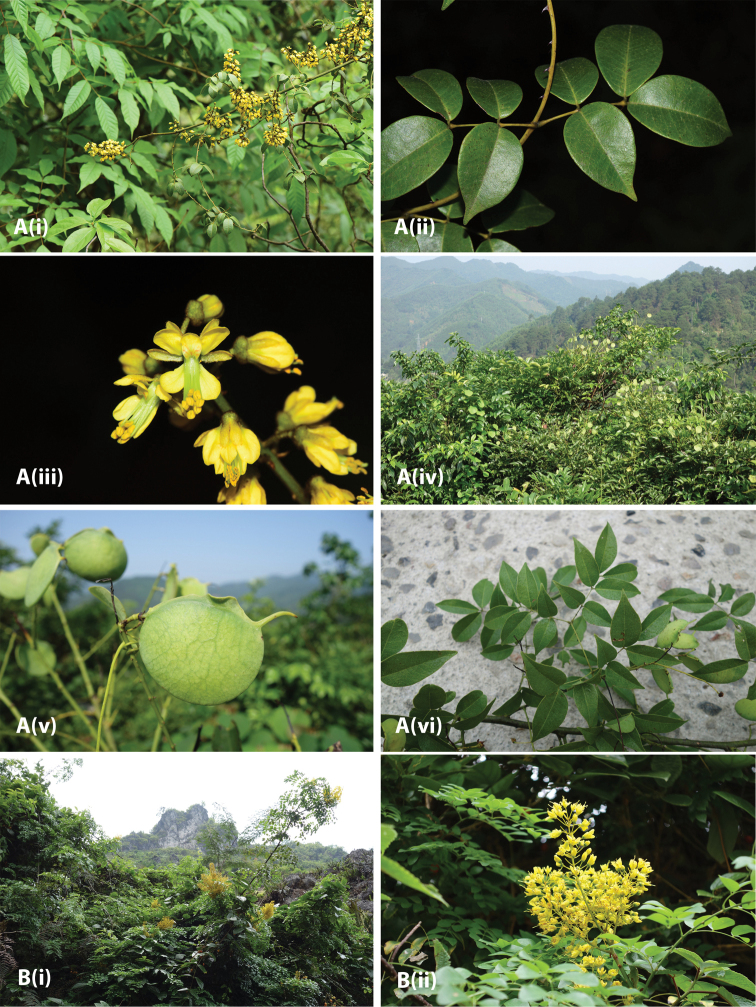
**A***T.sinensis* (i) habit (ii) leaves (iii) flowers (photos by Xin-Xin Zhu, Guizhou, China, *unvouchered*) (iv) habit (v) fruit (*R.P. Clark 429*, Guangxi, China, (IBK, K)) (vi) leaves (*R.P. Clark 415*, Guangxi, China (IBK, K) **B**T.aff.szechuenensis (i) habit (ii) inflorescence and leaves (*R.P. Clark 422*, Guangxi, China (IBK, K)).

***Habit*** a scandent shrub or vine to 13 m. ***Stems*** with scattered recurved prickles to 4 mm, glabrous or sparsely whitish to pale orange tomentose. ***Stipules*** persistent, triangular, 1–3 × 1–2.5 mm. ***Leaves*** with 2–4(–5) pairs opposite pinnae; petiole (1.3–)3–7 cm; leaf rachis 2.5–24 cm, with paired recurved prickles at pinna insertion points and scattered in between, sometimes densely; pinnae 2.5–12.5 cm, sometimes with recurved prickles in pairs at the leaflet insertion points and scattered in between; leaf rachis and petiole glabrous to sparsely whitish to pale orange tomentose; pinna rachis glabrous to sparsely pale orange tomentose; leaflets 2–5 opposite pairs per pinna; elliptic, base cuneate to rounded, sometimes oblique, apex usually acuminate, or acute, occasionally rounded; terminal leaflets 1.8–10.7 × 0.9–5.1 cm; lateral leaflets 1.9–9.2 × 0.8–4.7 cm; all leaflets glabrous on both surfaces or lower surface sparsely orange tomentose at base and on midvein, sometimes at margins, glossy above; venation reticulate, anastomosing. ***Inflorescence*** a panicle, axillary, supra-axillary or terminal, 7–42 cm long, axes sparsely to densely ferruginous tomentose, axis sometimes with small, recurved prickles; pedicels (3–)4–12(–17 in fruit) mm, articulated, sparsely to moderately ferruginous tomentose; bracts caducous, triangular, 0.5–2 × 1–1.5 mm; clusters of triangular scale-like bracts sometimes below base of raceme; bracteoles caducous, broad, elliptic, acute, 2–3 × 1–1.5 mm. ***Flowers*** with a hypanthium 1–2 × 3–5 mm, sparsely to moderately ferruginous tomentose; lower calyx lobe 6–8 × 3–5 mm, other lobes 5–6 × 2–3 mm, all lobes sparsely to densely pale orange to ferruginous tomentose on inner and outer surface; median petal obovate, sometimes reflexed backwards, inrolled, 6–8 × 3–4 mm, including claw 1–2 mm long, a circular patch of orange hairs between claw and blade, hairs on margins of claw; upper laterals obovate, 6–10 × 2–6 mm, including claw ca. 1 mm long, petal glabrous or with a few hairs on inner surface of claw; lower laterals 6–10 × 2–6 mm, including claw ca. 1 mm long, glabrous or with a few hairs on inner surface of claw; stamen filaments flattened, 5–12 mm long, densely orange villous on basal ½; anthers 1–2 mm long; ovary 2–5 mm long, sparsely to densely, sometimes partially, orange tomentose, occasionally glabrous; style 6–12 mm long, sparsely hairy on basal ½; stigma funnel-shaped, not or very slightly papillate, sometimes slightly laterally placed. ***Fruit*** light green, indehiscent, coriaceous, sub-circular to lunate, base cuneate to rounded, not stipitate or stipe 0–2 mm, apex with a pronounced beak to 25 mm, venation prominent, sparsely ferruginous tomentose, the indumentum most dense at base and on margins, glabrescent or glabrous, 3–5.8 × 1.9–3.6(–4.1) cm × ca. 4–8 mm deep, wing on ventral suture 0.5–4 mm wide. ***Seed*** 1, ca. 1.8–2.5 cm diameter, matt or glossy dark brown. (Fig. 3A).

##### Ecology.

Forest and thicket, on limestone. Elevation 100–1500m.

##### Phenology.

Flowering March-May, fruiting March-October.

##### Distribution.

China (Chongqing, Guangxi, Guangdong, Hong Kong, Hubei, Sichuan, Yunnan), Laos, Myanmar, Vietnam (Map 1).

##### Notes.

The current authors have not seen any specimens or specimen records from Hong Kong but the area is included within the distribution listed by Vidal and Hul Thol (1976) and that information is retained here. The identity of the few available specimens from Laos and Myanmar is unconfirmed and these could be individuals of *T.crista*, although based on morphological affinities and the preference of *T.crista* for coastal habitats they are retained here as *T.sinensis* pending further analysis of the species limits.

##### Selected specimens examined.

**China. Chongqing**: Qijiang, Wansheng, Heishangu Ave., opposite of Yaqulou, *S.R. Yi YSR9620* (NPH). Shimiaoxiangzhai, *S.G. Tang s.n.* (SM). Wushan, Guandu River, *T.P. Wang 10431* (WUK). **Fujian**: Mengtongyang, Chengmenkan, *H.Y. Zou 0931* (NF). **Guangdong**: Dinghu, Xinghu, Yuping Peak, *K.C. Ting & G.L. Shi 1337* (WUK). Zhaoqing, Qixingyan, *F.C. How 74128* (IBK). **Guangxi**: Bama, Xishan, *Z.T. Li 601739* (KUN). Donglan, Ma’an mountain, *R.P. Clark 429* (K, IBK). Debao, Longguang, Miaohuai Village, *Debao Exped. 451024160516009LY* (IBK). Fusui, Quli, Jidao Village, *B.Y. Huang*, *Y.Y. Xie & H.F. Cen 451421160523025LY* (GXMG). Jingxi, Longlin to Ande, *R.P. Clark 415* (K, IBK). Liuzhou Longtan Park, *W.E. Qun 150* (K). Long’an, Nanxu, Longxintun, *Long’an Exped. 450123130506007LY* (IBK). Longlin, Kechang, Haichang Village, Dankuntun, *L.Y. Yu*, *Y.D. Peng & X.Y. Hu 451031140410083LY* (GXMG). Napo, county town, Hongshui River Exped. 400 (TNM). Ningming, W Tingliang, *C.C. Huang et al. 2111* (GXMI). On the slopes of the limestone mountain near the county seat of Lingle, *Z.T. Li 603637* (IBK). S Nanning, Dar Shan, Seh-Feng, Me-Jon, *R.C. Ching 8435* (US). Tianyang, Wucun, Dalu Village, Longlitun, *Tianyang Exped. 451021150410060LY* (GXMG). **Guizhou**: Ceheng, Rongxian, Huangtian to Maoping, *Z.Y. Cao 544* (PE). Kwanlin, Kwanlinchow, Da-Swee-Tzi, *S.W. Teng 1641* (IBSC). Zhenfeng, Beipanjiang, Shuiyanba Village, *Y. Jia 522325190716483LY* (GZTM). **Hubei**: Badong, *T.P. Wang 10830* (IBK). **Sichuan**: Jiang’an, Nanyan, Hongfo Temple, *K.Y. Lang 3033* (PE). Xuanhan, Dong’an, *Xuanhan Exped. 1498* (SM). **Yunnan**: Between Szemao and Puerhfu, *J.F. Rock 2849* (NY, US). Lushui, near Nujiang River, *H. Sun 1672* (KUN). Xichou, Changqing, *C.W. Wang 81282* (KUN). Yingjiang, 23 km milestone on X309 Road from Pingyuan to Kachang, *Y.J. Guo*, *W.L. Zhao*, *P.X. Tang*, *X.L. Jin & X.Q. Zhang 13CS7525* (KUN). **Laos.** Phou Phung pres de Louang Prabang, *M. Poilane 20257* (K, L). **Myanmar.** Bhamo District, Lapycke to Sinlum Kabo, *J.H. Lace 5769* (K). **Vietnam.** Indo-China, Tonkin, *A. Petelot 4757* (NY). **Ninh Binh**: Cuc Phuong National Park, *N.M. Cuong 464* (MO).

#### ﻿7. Ticanto szechuenensis

Taxon classificationPlantaeFabalesFabaceae

(Craib) R. Clark & Gagnon comb. nov.

170B1EB5-BCCB-5C32-86A5-EC7E1679775F

urn:lsid:ipni.org:names:77303565-1

= Caesalpinia kwangtungensis Merr., J. Arnold Arbor. 8: 7. 1927; Herkl. in Hong Kong Naturalist ix. 32. 1938, descr. ampl. Type: China. Kwangtung, *Wilson in Canton Christ. Coll. 12838* (lectotype, designated here: (GH [A00059893!], isolectotypes: BM [BM000958803!], E [E00313522!], LU, NAS, [NAS00071304!, NAS00071305!], P [P02142689!], US [US00002578!]).

##### Basionym.

*Caesalpiniaszechuenensis* Craib, Pl. Wilson. (Sargent) 2(1): 92. 1914.

##### Type.

China. Western Szechuan, Kiating Fu, May 1908, *E.H. Wilson 3255*. (lectotype, designated here: K [K000980490], isolectotypes: A [A00059895!, A00059896!], BM [BM000958802!], E [E00313523!], GH, NY [NY00003572!], US.)

##### Description.

***Habit*** a scandent shrub. ***Stems*** with sparse, scattered recurved prickles, glabrous. ***Stipules*** minute, ca. 0.5 × 0.25 mm, triangular, subglabrous. ***Leaves*** with 3–6 pairs opposite to strongly subopposite pinnae; petiole 1.8–7 cm, leaf rachis with prickle at the base of each pinna and scattered in between pinnae insertions, 6–22 cm; pinnae 3.8–8.5 cm, occasionally with prickle at base of petiolule; leaflets 3–6 opposite pairs per pinna, elliptic to slightly ovate, the apex usually acute, occasionally slightly rounded; terminal leaflets 2–6 × 1–3 cm, lateral leaflets 1.2–6 × 0.8–3 cm, upper surface glabrous, lower surface glabrous or with a few ferruginous tomentose hairs on midvein at base or with sparse short ferruginous hairs; venation prominent on both surfaces, reticulate, anastomosing. ***Inflorescence*** a terminal, few-branched panicle, 11–15 cm long, axes subglabrous to sparsely to moderately ferruginous tomentose; pedicels (5–)9–11 mm, articulated, glabrous to sparsely ferruginous tomentose; bracts not seen; bracteoles caducous, 1 × 0.25 mm, lanceolate-acuminate. ***Flowers*** with a hypanthium ca. 1–2 × 3–4 mm, sparsely ferruginous tomentose; lower calyx lobe 8 mm long, other lobes 5 mm long; median petal 6–9.5 × 2.5–5 mm, with a patch of hairs at base of blade and few hairs on the claw; upper laterals ca. 5–10 × 3–4 mm, glabrous or with a few hairs on inner surface of the claw; lower laterals ca. 5–10 × 3–4 mm, glabrous or with a few hairs on inner surface of claw; stamen filaments flattened, ca. 9 mm long, densely orange villous on basal ½; ovary ca. 2.5 mm long, sparsely to moderately densely pale orange tomentose; style 10–12 mm, with a few hairs at the base, otherwise glabrous, ovules 2; stigma funnel-shaped, papillate, ca. 1 mm wide. ***Fruit*** indehiscent, coriaceous, strongly asymmetrical, sub-lunate to sub-circular or teardrop-shaped, stipe 0–1 mm, beak 1–5 mm, venation prominent, glabrous, 1.5–3.4 × 1.5–3 cm × 0.4–0.6 cm, wing sometimes present along part of length of ventral suture, 1–3 mm wide. ***Seed*** 1, circular, dark brown, 1.4–1.7 cm diameter (Fig. 3B).

##### Ecology.

Mountain forest, thicket, on limestone, elevation 260–1500 m.

##### Phenology.

Flowering April-August, fruiting June-October.

##### Distribution.

China (Chongqing, Fujian, Guangdong, Guangxi, Hong Kong, Hunan, Sichuan) (Map 1).

##### Notes.

The current authors have seen no specimens or specimen records from Hong Kong and inclusion of the species in that area follows Vidal and Hul Thol (1976).

##### Selected specimens examined.

**China. Chongqing**: Jiangjin, Mt. Simianshan, Sunzigang, *Z.Y. Liu*, *J. Zhang et al. S-2006* (IMC). Nanchuan, Mt. Jinfoshan, Sanquan, Lengshuixi, *Z.Y. Liu 960468* (IMC). **Fujian**: Yunxiao, Xiaban, Mt. Dachenshan, *G.D. Ye 2038* (PE). Zhangzhou, Yunxiao, Mt. Liangshan, Yunliang Reservoir, *X.F. Zeng ZXF36083* (CZH). **Guangdong**: Gaoyao, at the foot of Mt. Dinghushan, *C. Huang 161752* (IBSC). Ruyuan, Daqiao, *Yue71 466* (IBSC). **Guangxi**: Jingxi to Longbang, *R.P. Clark 422* (K, IBK). Liuzhou, Rongan, Banqiao, Guban Village, *Rong’an Exped. 450224170806001LY* (GXMG). **Hunan**: Yizhang, Changle, Mt. Xinpingshan, *S.K. Lau 29560* (IBSC). Yongzhou, Jiangyong, Lanxi, Shangjin Village, *X.C. Jiang*, *G.H. Tang & X.W. Pan SCSB-HNJ-0051* (KUN). **Sichuan**: Changning, Xiangling, Liushuiyan, *s. coll. 704* (SM). Gongxian, Luobiao, Wangjia, *s. coll. 278* (SM). Hongya, Liujiang, Shuguang, Laoyingzui, *Hongya Group 420* (SM). Junlian, Tuanjie, Lüzhu Temple, *Sichuan Economic Plants 0281* (PE). Leibo, Zhongshanping, Xining, *Sichuan Economic Plants 487* (CDBI). Mt. Emei, Heilongjiang, *K.T. Fu 12134* (WUK). Pingshan [Ping-shan], *F.T. Wang 22721* (PE). Tongliang, Xiquan, Xiafeng, *Tongliang Exped. 267* (SM). Xuyong, Shuiwei, Guandou Village, across the Qiaogoutou River, *X.F. Gao*, *Y.D. Gao & W.B. Ju HGX10640* (CDBI).

#### ﻿8. Ticanto vernalis

Taxon classificationPlantaeFabalesFabaceae

(Champion ex Benth.) R. Clark & Gagnon comb. nov.

40460AC2-9457-59AF-A3DE-A438B24E3327

urn:lsid:ipni.org:names:77303566-1

##### Basionym.

*Caesalpiniavernalis* Champion ex Benth., Hooker’s J. Bot. Kew Gard. Misc. 4: 77. 1852.

##### Type.

China. Hong Kong, *Champion in Herb. Bentham 502* (neotype (designated by Vidal and Hul Thol 1976): K [K000789359!])

##### Description.

**Figure 4.**
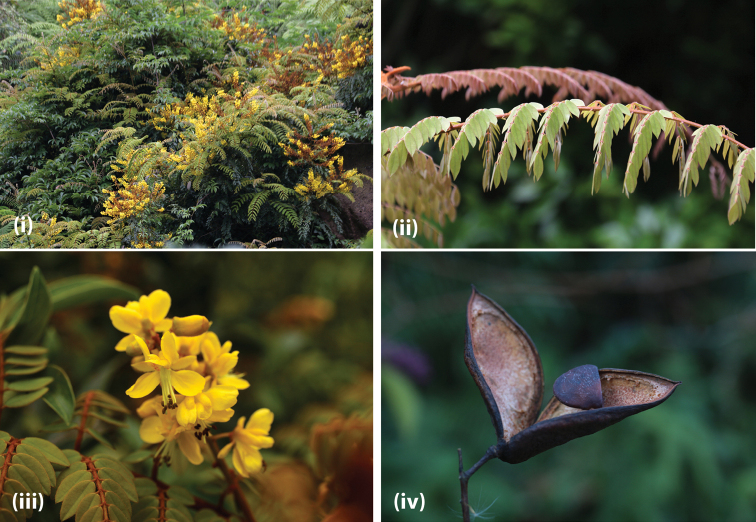
*T.vernalis* (**i**) habit (**ii**) leaves (**iii**) flowers (**iv**) fruit (photos by Jian-Ping Wu, Zhejiang, China, *unvouchered*).

***Habit*** a liana. ***Stems*** moderately to densely ferruginous tomentose, glabrescent when old, sometimes with scattered recurved prickles. ***Stipules*** triangular, 1–2 × ca. 1 mm. ***Leaves*** with 8–16 pairs opposite to strongly subopposite pinnae; petiole 1–2.5 cm; rachis 20–43 cm long, with a recurved prickle at the base of each pinna and scattered along the rachis between the pinnae insertions, moderately to densely ferruginous tomentose; pinnae 4.5–8 cm; leaflets 5–10 opposite pairs per pinna, coriaceous, elliptic to ovate, apex acute, mucronulate, terminal leaflets 1.4–2.8 × 0.5–1.5 cm, lateral leaflets 1.2–2.5 × 0.5–1.3 cm, both surfaces glabrous, or lower surface sparsely ferruginous tomentose, or only on midvein; venation reticulate, anastomosing, obscure. ***Inflorescence*** a raceme or many-branched panicle 12–35 cm long, in axils of upper leaves or terminal, axes and pedicels densely ferruginous tomentose; bracts not seen, bracteoles ca. 1–2 × 1 mm, apex acuminate, sparsely to densely ferruginous tomentose; pedicels 6–12(–16 in fruit) mm. ***Flowers*** with a hypanthium ca. 2 × 4 mm, this moderately to densely ferruginous tomentose; lower (cucullate) lobe ca. 7–11 × 4 mm, sparsely to moderately ferruginous tomentose on centre of outer surface, becoming glabrous towards the edges, other lobes ca. 6–12 × 2 mm, (very) sparsely ferruginous tomentose inner and outer surfaces sparsely ferruginous tomentose; median petal (6–)9 × 2 mm, inrolled, with dense circular patch of hairs at base of blade, and some hairs on claw, particularly on the margins; upper laterals ca. 9 × 3 mm, sparsely tomentose on inner surface of claw; lower laterals ca. 10–11 × 3 mm, sparsely tomentose on inner surface of claw; stamen filaments flattened, ca. 9–12 mm, pale orange tomentose on lower ca. ^2^/_3_ on inner surface; anthers ca. 1.5–2 mm long; ovary ca. 2.5 mm long, densely ferruginous tomentose, stipe ca. 1 mm long, style ca. 6 mm, glabrous, ovary 2-ovuled; stigma funnel-shaped, slightly papillate, ca. 1 mm wide. ***Fruit*** dehiscent, ligneous, obliquely oblong or sub-elliptic, slightly asymmetrical, apex beaked, venation obscure, sparsely to densely ferruginous tomentose, 4–6 × 2.5–4 × 1–1.3 cm, ventral suture lacking a wing. ***Seeds*** (1–)2, lunate, ca. 2.1–2.7 × 1.3–2.1 cm, matt black (Fig. 4).

##### Ecology.

Moist sandy soils, beside rocks along valleys, in thickets; elevation ca. 600 m.

##### Phenology.

Flowering February-April, fruiting September-December.

##### Distribution.

China (Fujian, Guangdong, Hong Kong, Zhejiang) (Map 1).

##### Selected specimens examined.

**China. Fujian**: Hua’an, Eshan, *W.D. Han 20542* (NF). Yunxiao, Datian, *G.D. Ye s.n.* (FJSI). **Guangdong**: Baoan, Shatian, *T. Chung M185* (IBSC). Chaochow, Raoping, Fenghuangshan, *N.K. Chun 42662* (IBSC). Guangzhou, Conghua, Daling, Shanshizao, *S.J. Li 787* (IBSC). Haifeng, Lianhua, Lügong, Dakeng, *G.X. Chen 24* (IBSC). Huidong, *Z.Q. Song 2021061* (IBSC). Luofushan, Sulao Taoist Temple, on the way to Dachashan, *Y. Tsiang 1751* (IBSC). Meizhou, Fengshun, Yanping, Fengbei Village, *X.F. Zeng ZXF01805* (CZH). **Hong Kong**: Lantau, Tung Chung, *S.Y. Hu 12897* (PE). N. T. Lan Nai Chung, Sai Kong, *S.Y. Hu 8570* (PE). **Zhejiang**: Huangyan, Western part, Shidun, *N.Z. Wang s.n.* (NAS). Jiande, Jiande Forest Farm, *J. Zhao et al. 8524205* (PE). Jingning, Wangkeng, *M.L. Yu et al. 25125* (HHBG). Jiansae, Laufuyoh, *K.W. Jiang*, *J.P. Wu*, *Y.F. Zhang*, *M.S. Zhang et al. YS022* (NPH). *ibid.*, *Y.M. Zhang YS023* (IBSC). Linhai, Yongdongkou, Dakeng, *s. coll. 196* (HHBG). Ningpo, Tientungssu, *H. Migo s.n.* (NAS). Suichang, Daban, Yakoumen, *M.L. Yu 25756* (NAS). Tiantai, Gaoming, *L.S. Que 28517* (ZM). Wencheng, Shuiyanhu, *J.P. Feng 499* (HHBG). Yueqing, Dajing, Dianling, Dazhuyuan, mountainside, *Hangzhou Botanical Garden Herbarium 2493* (HHBG).

#### ﻿9. Ticanto yunnanensis

Taxon classificationPlantaeFabalesFabaceae

(S. J. Li, D. X. Zhang & Z.Y. Chen) R. Clark & Gagnon comb. nov.

901D8FF3-AB92-5CA5-92C2-353394F41EF9

urn:lsid:ipni.org:names:77303567-1

##### Basionym.

*Caesalpiniayunnanensis* S. J. Li, D. X. Zhang & Z.Y. Chen. Novon 16(1): 78–80. 2006.

##### Type.

China. Yunnan, Xishuangbanna, *T. P. Zhu* (*Zhu Tai-Ping*) *139* (holotype KUN: [1206956!], isotype IBSC [0162107!]).

##### Description.

***Habit*** a liana. ***Stems*** with recurved prickles to ca. 2 mm long, glabrous. ***Stipules*** caducous, not seen. ***Leaves*** with 3 pairs opposite pinnae, petiole 7–8 cm, rachis 15–20 cm long, rachis with recurved prickles at the base of each pinna and scattered in between the pinnae insertions, pinnae 5–6.5 cm long; leaflets 2–3 opposite pairs per pinna, blade coriaceous, elliptic or narrowly elliptic, base obtuse to cuneate, apex bluntly acuminate, margin incurved abaxially, 6–11.5 × 2.5–4.5 cm, upper surface glossy, lower surface dull, both surfaces glabrous, 2^o^ venation anastomosing, 3^o^ venation finely reticulate. ***Inflorescence*** an axillary raceme, more than 20 cm long; pedicels ca. 7 mm. ***Flowers*** unknown; receptacle remnant ca. 3 mm wide. ***Fruit*** dehiscent, ligneous, oblong to elliptic, slightly asymmetrical, base widely cuneate, apex with beak ca. 2 mm long, venation reticulate, not prominent; 5–7 × 2.8–3.5 cm, ventral suture lacking a wing. ***Seed*** 1.

##### Ecology.

Thickets along riversides, sparse woodlands along roadsides, elevation ca. 600 m.

##### Phenology.

Flowering unknown, fruiting October.

##### Distribution.

China (Yunnan) (Map 1).

##### Notes.

Specimen *Chen 0066* which is listed as a paratype of *T.yunnanensis* has leaflets that are asymmetrical, with an asymmetrical base and distinctly acuminate tip, and the secondary veins are at a more acute angle to the midvein when compared with typical *T.yunnanensis*. It is sufficiently morphologically divergent from the concept of *T.yunnanensis* to be considered by the current authors to represent a different taxon (not determined), and the characters of that specimen are therefore not included in the description above.

##### Selected specimens examined.

**China. Yunnan**: Xishuangbanna, Mengla, Yiwu, *J.H. Zhang 19335* (HITBC).

### ﻿Excluded names

*Guilandinarotunda* Noronha, Verh. Batav. Genootsch. Kunst. 5(Art. 4): 16 (1790), *nom. inval. nom. nud*.

*Butealoureiroi* Spreng., Syst. Veg., ed. 16(3): 186. 1826. *nom. inval. nom. superfl*.

*Caesalpiniascandens* J. Koenig ex Baker in Hook. F., Fl. Brit. India 2(5): 255 (1878), *nom. inval*.

### ﻿Fossil taxa

Although there are no fossils definitively attributed to *Ticanto*, some partial leaf fossils from the Upper Tochiwara Formation of Japan, dating to the mid Miocene, have been tentatively likened to *Caesalpiniacrista* and assigned the name *Caesalpiniahokiana*Ozaki (1980). However, it is not clear from the text of that publication, or the fossil material itself, whether the *C.crista* under comparison is the species here included in *Ticanto* or the alternative species to which that name is commonly (though erroneously) applied, *Guilandinabonduc* L. The fossil material is also compared with *Caesalpiniajaponica* Siebold & Zucc. (= *Biancaeadecapetala* (Roth) O. Deg.), but is in fact inadequate to be attributed confidently even to *Caesalpinia* s.l. No combination for *Caesalpiniahokiana* is therefore made here in *Ticanto*, but the possibility that the species belongs within the genus cannot be eliminated.

### ﻿Nomenclatural notes on Caesalpinia (Ticanto) crista

The protologue of *Caesalpiniacrista* L. lists three type elements: *Fl. Zeyl.* 157; *Pluk. alm.* 4. *t.* 2. *f.* 2.; *Breyn. ic.* 58. *t.* 28. Of these, only the *Flora Zeylandica* element references material (in Hermann's Herbarium) attributable to *Caesalpiniacrista* L. whilst the Plukenet figure and Breynius plate are representative of *Guilandinabonduc* L. (1753) (see Dandy and Exell 1938). This has been a source of nomenclatural confusion, resulting in the name *Caesalpiniacrista* being erroneously applied to *G.bonduc*. In addition, names synonymous with *G.bonduc* L. have been mistakenly placed in synonymy with *C.crista* L. by various authors. Adding to the confusion, the name *Guilandinacrista* (L.) Small was based on the name *Caesalpiniacrista* L., and is therefore a synonym of that name, although the protologue of *G.crista* clearly describes a species of *Guilandina* (features include large, prickly fruits, grey seeds, and distribution including the Florida Keys).

The impact of the application of the name *Caesalpiniacrista* to two widespread species, both of which have medicinal uses, is significant. A wide range of studies record the presence of numerous phytochemicals in *C.crista* along with a wealth of reported pharmacological benefits including antioxidant, antibacterial, antiviral (including for treatment of Covid-19 [Ahmad et al. 2021]), anti-malarial, anti-tumour, anticancer, anti-diabetic, anti-inflammatory, analgesic, hepatoprotective, cardioprotective, anti-amyloidogenic, nootropic, wound healing, anthelmintic, insecticidal, antipyretic and antiulcer activities (Ramesh et al. 2010; Sarkar et al. 2012; Kumar et al. 2017; Chan et al. 2018; Chethana et al. 2018; Srinivasa et al. 2019; Amirtharaj and Sivashankar 2021). These apparent benefits reflect long-standing and diverse traditional medicinal uses in countries including India, Myanmar, Sri Lanka and Indonesia of the so-called ‘fever nut’, a name often used for the species *Guilandinabonduc* L. (also known as nickar bean or grey nickar bean). Although the majority of published phytochemical and pharmacological analyses referring to *Caesalpiniacrista* appear to concern the species *Guilandinabonduc* L., a review of these studies reveals widespread lack of clarity surrounding the identity of the taxon under study. Details of the material under investigation include mixed lists of synonyms, species descriptions that appear to combine elements of the morphology of both *C.crista* and *G.bonduc*, or photos of *C.crista* presented to represent studies of *G.bonduc* (Chan et al. 2018; Upadhyay et al. 2019). Several studies lack reference to a voucher specimen (although the material is usually said to have been verified by a botanist; Gupta et al. 2013; Sharma et al. 2013; Chauhan et al. 2015; Srinivasa et al. 2019), or the voucher reference is incomplete (Yang et al. 2009; Kumar et al. 2017) and thus the identity of the material cannot be easily verified. Most studies that cite a voucher specimen do not present photographs of the plant material used (Kalauni et al. 2004; Cheenpracha et al. 2005; Linn et al. 2005; Patil 2005; Awale et al. 2006; Jabbar et al. 2007; Satnami and Yadava 2011; Sarkar et al. 2012; Chethana et al. 2018; Amirtharaj and Sivashankar 2021). If provided these may be too poor to enable identification (Upadhyay et al. 2019), and digital images of the voucher specimens are rarely available online. Confusion of the species’ identity is also apparent on web resources such as the India Biodiversity Portal (https://indiabiodiversity.org), Tropical Plants Database (http://tropical.theferns.info/), Mangroves of Odisha (https://odishamangroves.in/) and those describing or retailing traditional medicine (e.g., https://ayushvedah.com/, https://www.planetayurveda.com/) which provide synonyms and photos of *C.crista* alongside descriptions of *G.bonduc*. Other resources present details of true *Caesalpiniacrista* (e.g., Flora and Fauna Web, a Singapore Government agency website, https://www.nparks.gov.sg/) without reference to the common, though erroneous, application of the name to *G.bonduc*. Compounding the nomenclatural issue between the two species is the morphological similarity between them (both are prickly, scrambling shrubs with yellow generalised caesalpinioid flowers and fruit with 1–2 seeds), and by their mutual tendency to inhabit coastal areas.

The implications of misidentification of samples used to test for the presence of biologically active phytochemicals and to evaluate medicinal properties are clear. Taxonomic uncertainty could lead to false assumptions of the properties of a species or inclusion of the wrong species in medical preparations, and potential harm to human health. Reiteration of the correct application of the name *Caesalpiniacrista* L. provided here will contribute to avoidance of this issue in future studies.

## ﻿Discussion

### ﻿Recognition of *Ticanto*

Genera segregated from *Caesalpinia* s.l. are most often morphologically characterised by fruit and floral characters, as well as by glands and trichomes (Gagnon et al. 2016). However, certain genera recognised by Lewis (2005) including *Caesalpinia* s.s., *Denisophytum* and *Erythrostemon*, and *Arquita*, recognised by Gagnon et al. (2015), were recircumscribed by Gagnon et al. 2016 to reflect true phylogenetic relationships, and these have been more difficult to define morphologically. Nonetheless, recognition of these genera is justified given that the alternative would be to continue to recognise a massive Caesalpinia Group that also lacks clear morphological diagnostic characters to distinguish it from other groups and genera in the Caesalpinieae tribe.

Gagnon et al. (2016) refrained from reinstating the genus *Ticanto* because of the limited amount of sampling in their phylogeny of this genus. Furthermore, no diagnostic synapomorphies were identified to distinguish the species provisionally ascribed to *Ticanto* from the genera to which they are most closely related, *Pterolobium*, *Mezoneuron* and *Biancaea*. It was noted that two species putatively placed in *Ticanto* (*Caesalpiniacaesia* and *Mezoneuronsinense*) sometimes have a winged fruit, but that character also occurs in these three related genera. However, the phylogenies reconstructed here show that by sampling multiple species that were putatively attributed to this group, a strongly supported monophyletic clade is recovered, (bootstrap = 94%, PP = 1.0) sister to the long-recognised genus *Pterolobium* (Fig. 1). The very robust values supporting the clades containing *Ticanto* species and the most closely related genera provide strong justification for recognising *Ticanto* as a distinct genus. Our thorough revision of the group also allows us to verify that the group is morphologically distinct from the most closely related genera.

The morphological resemblance of species now included in *Ticanto* was detected as long ago as the 19^th^ century, when Prain (1897) suggested that *Caesalpinianuga* (= *T.crista*) and *Mezoneuronsinense* (= *T.sinensis*) may be better placed together in a proposed new genus, *Nugaria*. Later, Clark (2016) observed the similarity of fruits of *Mezoneuronsinense* to those of five species here attributed to Ticanto, and commented on the possible generic misplacement of that species in *Mezoneuron*. Although there are no unique morphological synapomorphies to define *Ticanto* as circumscribed in this account, the species are united by a combination of characters: the lianescent or scrambling habit, armature of recurved prickles, a pari-bipinnate leaf, a laterally compressed fruit with 1(–2) seeds that is usually indehiscent and is with or without a narrow sutural wing, and relatively small, unspecialised flowers. The capacity of each of these features to diagnose the genus will now be discussed.

The vegetative characters of *Ticanto* do not distinguish it clearly from *Pterolobium*, *Mezoneuron* and *Biancaea*, all being lianas or scandent or trailing shrubs armed with recurved prickles, and with pari-bipinnate leaves (Fig. 2, 3, 4). However, some divergence can be observed between the leaves of *Ticanto* and those of *Pterolobium* in that *Ticanto* usually bears few pinnae (1–8 pairs, although up to 16 pairs in *T.vernalis*) and few, relatively large leaflets (2–10 pairs, or up to 15 in *T.caesia*), whereas *Pterolobium* is characterised by numerous pinnae (4–20 pairs) and numerous, small leaflets (4–25 pairs per pinna). The leaflets of *Ticanto* are usually elliptic or rhombic (except *T.caesia*) whilst those of *Pterolobium* are oblong, strongly asymmetric at the base, and regular in size. It should be noted, however, that these differences are tendencies rather than discrete states.

The flowers of all four genera can be considered as ‘typical’ Caesalpinia group flowers consisting of a short hypanthium and five sepals, the lower of which is cucullate over the others in bud, and five oblong, obovate or spathulate (occasionally bilobed) petals that are yellow or white, sometimes with red markings on the median petal, usually with some degree of pubescence. Stamens are free and tomentose, the ovary is glabrous or hairy, and the stigma is cupular, funnel-shaped or truncate, often papillate. The flowers of all four genera appear to be adapted for a range of generalist pollinators (mostly species of bee) and do not exhibit modifications related to novel pollination syndromes (although ambophily, i.e., pollination by both wind and insects, is reported uniquely in *T.crista* by Li et al. 2004), which seems to result in an absence of generic level floral distinctions among the four similar genera.

**Figure 5.**
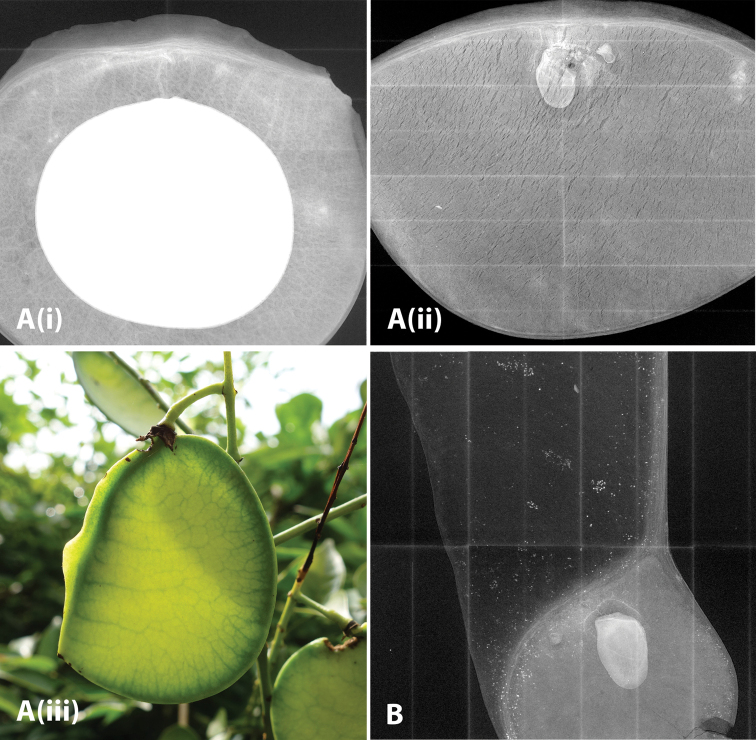
Fruit wing venation **A***T.sinensis* fruit with wing (i) (X-ray) (*Z. Zhang 148*, China (MO)) (ii) (X-ray) (*W.E. Qun 150*, China (K)) (iii) (photo by R.P. Clark, Guangxi, China, *unvouchered*) **B***Pterolobiummicrophyllum* fruit with wing (X-ray) (*C. Phengkhlai 682*, Thailand (K)).

Like several other genera in the Caesalpinia group, *Ticanto*, and the genera to which it is most closely related (*Pterolobium*, *Mezoneuron* and *Biancaea*) are distinguished primarily by differences in fruit morphology. The fruits of *Ticanto* are elliptic, circular or lunate, compressed or inflated, coriaceous or ligneous, usually indehiscent (two dehiscent exceptions), 1–2-seeded, and with or without a narrow wing up to 4 mm wide along the upper suture (Fig. 5) (or a carinate wing 5–6 mm deep) (Table 3). The genus *Mezoneuron*, sister to the *Ticanto*-*Pterolobium* clade, is characterised by fruits that are elliptic to oblong, laterally compressed, chartaceous to coriaceous, indehiscent, with a wing along the upper suture 2–20 mm wide, containing one to 13 seeds (11 species multi-seeded, nine species single-seeded, three unknown). There is thus a degree of congruence between the fruits of *Ticanto* and those of *Mezoneuron* with respect to indehiscence, number of seeds and presence of a wing (variable in *Ticanto*, universal in *Mezoneuron*). However, the majority of *Mezoneuron* fruits are multi-seeded and the wing is usually broader than 3 mm, whilst the species of *Mezoneuron* bearing single-seeded and narrow-winged fruit most like those of *Ticanto* are distributed in Australia, New Caledonia and New Guinea and are thus allopatric with respect to almost all species of *Ticanto* (except *T.crista*).

**Table 3.** Comparative characters of *Ticanto* species (characters considered to be most taxonomically informative are in bold).

	*T.caesia*	*T.crista*	*T.elliptifolia*	*T.magnifoliolata*	*T.rhombifolia*	*T.sinensis*	*T.szechuenensis*	*T.vernalis*	*T.yunnanensis*
**Pairs pinnae**	**5–8(–9)**	**3–6(–8)**	**1–2**	**2–3(–4)**	**3–6**	**2–4(–5)**	**3–6**	**8–16**	**3**
**Position of pinnae**	opposite	opposite (occasionally slightly subopposite)	opposite	opposite	opposite	opposite	opposite (rarely subopposite)	opposite to strongly subopposite	opposite
**Pairs leaflets**	**8–15**	**2–4(–7)**	**2**	**2–3**	**3–5**	**2–5**	**3–6**	**5–10**	**2–3**
**Leaflet size**	**0.8–1.5 × 0.4–0.6**	**2.1–7.2 × 1–3.3**	**7–13 × 4.5–8**	**3.5–10.8(–15) × 2.1–7**	**1.5–2 × 0.8–1.3**	**1.8–10.7 × 0.8–5.1**	**1.2–6 × 0.8–3**	**1.2–2.8 × 0.5–1.5**	**6.0–11.5 × 2.5–4.5**
**Leaflet apex**	**truncate, obtuse, rounded, emarginate**	**obtuse or rounded to retuse, acute, or acuminate**	**rounded, obtuse or acute**	**usually obtuse, rounded, retuse or emarginate, occasionally acute**	**acute**	**usually acuminate or acute, occasionally rounded**	**usually acute, occasionally slightly rounded**	**acute**	**bluntly acuminate**
**Inflorescence indumentum**	brown puberulent	glabrous or sparsely tomentose	densely hairy	sparsely to moderately tomentose	glabrous or subglabrous	sparsely to densely ferruginous tomentose	subglabrous to moderately tomentose	densely tomentose	unknown
**Inflorescence length cm**	10–15	8–40	unknown	15–30	10–20	7–42	11–15	12–35	>20
**Pedicel length mm**	4–7	5–15	8–12	5–11	6–9	3–12(–17 in fruit)	5–11	6–12(–16 in fruit)	ca. 7
**Bracteoles mm**	unknown	1 – 2.5 × 0.5 – 1	unknown	unknown	unknown	broad, 2–3 × 1–1.5	caducous, 1 × 0.25 mm, lanceolate-acuminate	1.2 × 1	unknown
**Median petal size mm**	3.5–5.5 (L)	6–9 × 3–7	ca. 10.5 × 6–7	ca. 7–10 × 3–5	ca. 7 (L)	6–8 × 3–4	6–9.5 × 2.5–5	6–9 × 2	unknown
**Lateral petals size mm**	3.5–5.5 (L)	6–11 × 3–6	10–12 × 4–5	ca. 7–10 × 3–5	ca. 7 (L)	6–10 × 2–6	5–10 × 3–4	9–11 × 3	unknown
**Median petal indumentum (inner surface)**	rhombic patch of hairs	dense circular patch of hairs in middle	circular patch of hairs	patch of hairs	patch of hairs	circular patch of orange hairs in middle, hairs on margins of claw	patch of hairs in middle; few hairs on margins of claw	dense circular patch of hairs in middle, some hairs on claw	unknown
**Stamen length mm**	ca. 6	4 – 12	9–14	5–10	7	5–12	ca. 9	9–12	unknown
**Style length mm**	ca. 4	ca. 4–11	7–10	ca. 5–10	10	6–12	10–12	6	unknown
**Ovary indumentum**	**glabrous**	**glabrous or sparsely or patchily hairy**	**tomentose**	**glabrous**	**glabrous**	**sparsely to densely tomentose or glabrous**	**sparsely to moderately tomentose**	**densely pilose**	unknown
**Fruit dehiscence**	**indehiscent**	**indehiscent**	**indehiscent**	**indehiscent**	**indehiscent**	**indehiscent**	**indehiscent**	**dehiscent**	**dehiscent**
**Fruit size cm**	**4.5–5 × 2.3–5**	**2.7–7 × 2.2–3.7**	**4.5–5 × 2.2–2.5**	**2.8–4.2 × 2.2–3.5**	**3–4 × 2.3–2.5**	**3–5.8 × 1.9–4.1**	**1.5–3.4 × 1.5–3**	**4–6 × 2.5–4 × 1–1.3**	**5–7 × 2.8–3.5**
**Fruit wing**	**narrow**	**absent**	**absent**	**carinate, 5–6 mm**	**absent**	**0.5–4 mm wide**	**sometimes present, 1**–**3 mm wide, along part of fruit length**	**absent**	**absent**
**Fruit shape**	**elliptic**	**elliptic to lunate, sub-symmetrical to somewhat asymmetrical**	**oblong-elliptic, sub-symmetrical to sub-lunate**	**lunate**	**asymmetrical, sub-lunate**	**Sub-circular to lunate**	**strongly asymmetrical, sub-lunate to sub-circular or teardrop-shaped**	**obliquely oblong or sub-elliptic, slightly asymmetrical**	**oblong to elliptic, slightly asymmetrical**
**Fruit stipe mm**	unknown	2–5	short	ca. 1	ca. 3	0–2	0–1	unknown	unknown
**Fruit beak mm**	unknown	1–10	1–5	2–7	slight beak	pronounced beak to 25	1–5	beak present	ca. 2
**Fruit venation**	prominent	prominent	prominent	prominent	prominent	prominent	prominent	not prominent	not prominent
**Fruit indumentum**	glabrous	glabrous or very sparsely tomentose	unknown	glabrous	glabrous	glabrous or glabrescent	glabrous	sparsely to densely tomentose	unknown
**Fruit texture**	ligneous	coriaceous	coriaceous	coriaceous	coriaceous	coriaceous	coriaceous	ligneous	ligneous
**Ecology & elevation**	forests along rivers, 200–1000 m	often coastal; limestone, up to 350 m	ca. 100 m	forests, 400–1800 m	thickets	forest, thicket, limestone, 100–1500 m	forest, thicket, limestone, 260–1500 m	Moist sandy soils, thickets, ca. 600 m	unknown

The fruits of *Pterolobium* bear some structural similarities to those of *Ticanto* in that they comprise a 1(–2)-seeded locule that is always (*Pterolobium*) or sometimes (*Ticanto*) winged, and the wing is non-vascularised (no venation is conspicuous on the exterior surface or in X-ray, Fig. 5). However, the fruits of *Pterolobium* are unique within the Caesalpinia group in being samaroid, with a basal seed chamber. The surface of the fruit wing of *Pterolobium* also differs from that of *Ticanto* in having sculpturing in the form of parallel lines (the surface is smooth in *Ticanto*). The distinctiveness of the fruits of *Pterolobium* compared with all others in the Caesalpinia group, and particularly compared with those of *Ticanto*, strongly supports the recognition of two genera to represent those clades.

The fruits of *Biancaea* differ from *Ticanto* in that they are usually dehiscent and wingless (except *B.decapetala* which may have a narrow wing or ridge along the upper suture, and *B.millettii* which may have a very narrow wing along the upper suture) whilst those of *Ticanto* are usually indehiscent (with the exceptions of *T.vernalis* and *T.yunnanensis*) and often with a narrow wing along the upper suture, although some species are wingless (*T.crista*, *T.elliptifolia*, *T.rhombifolia*, *T.vernalis*, *T.yunnanensis*). The ovary indumentum of *Biancaea* is (densely) hairy, as opposed to glabrous or sparsely to (less commonly) densely hairy in *Ticanto*. The fruits of *Biancaea* are 2–8-seeded (apart from *B.millettii* with 1 seed), versus 1(–2)-seeded in *Ticanto*. *Biancaea* usually has large stipules, ranging from 3 mm to 4.5 cm long (except for *B.millettii* in which they are 2 mm long), whilst those of *Ticanto* are 0.25–3 mm long. *Biancaeamillettii* has morphological affinities with *Ticanto* in having small stipules and single-seeded fruits that sometimes have a narrow wing, and its distribution (Guangdong, Guangxi, Hunan, Jiangxi in China) is congruent with the centre of diversity of *Ticanto*. The leaves of *B.millettii*, which bear numerous pinnae and numerous, small, oblong leaflets, resemble those of *Ticantocaesia*. The morphological affinities of *B.millettii* with *T.caesia* (including certain fruit characters), and the distribution of the former in southern China (the centre of diversity of *Ticanto*), raised the question of whether *B.millettii* might belong in *Ticanto*; however, the molecular phylogenetic analysis here presented demonstrates it to be correctly placed in *Biancaea*. The fruit of *B.millettii*, despite some similarity with *Ticanto* fruits, exhibits features typical of *Biancaea* fruits in being dehiscent and with a puberulent indumentum. This supports the hypothesis that fruit characters are important in delineating segregate genera of *Caesalpinia* s.l.

### ﻿Geographical distribution of *Ticanto*

The distribution of *Ticanto* compared with closely related genera suggests it to be a distinct evolutionary lineage. The centre of diversity of *Ticanto* is Southern China, where all species are present and six are endemic (*T.caesia*, *T.elliptifolia*, *T.magnifoliolata*, *T.szechuenensis*, *T.vernalis*, and *T.yunnanensis*). Of the three remaining species, *T.rhombifolia* occurs also in northern Vietnam, *T.sinensis* extends into northern Laos, Myanmar and Vietnam, and only *T.crista* is more widely distributed throughout South-East Asia. The centre of diversity of its sister genus *Pterolobium* is South-East Asia (and a single species in Africa) from India through Myanmar into Indochina, and to Indonesia, Borneo, the Philippines, and Malaysia. Only two species of *Pterolobium* are found in China, namely *P.macropterum* Kurz, in Yunnan and Hainan provinces, and *P.punctatum* Hemsl., which is a broadly distributed Chinese endemic extending into at least nine provinces (International Legume Database and Information Service, https://ildis.org/LegumeWeb/). The distribution of *Ticanto* reflects its preferred ecological niche, characterised by a drier and more seasonal climate and scrub or dry forest habitat, and that of *Pterolobium* likewise reflects its preference for a warmer, moister climate and lowland forest habitat. *Mezoneuron* occurs (like *Pterolobium*) predominantly in the moist tropics, but with a more widespread and disjunct distribution (across South East Asia, two species in Africa, six species endemic to New Caledonia, one endemic in Madagascar, and one endemic in Hawaii), with only two species (*M.cucullatum* and *M.enneaphyllum*) present in southern China, represented by just a few specimens collected close to the borders. Of the six species in the Asian genus *Biancaea*, *B.millettii* is endemic to a few provinces in southern China, whilst the widely distributed *B.decapetala* is present throughout southern China, and the remaining four species occur in moist tropical areas from India to Thailand, Cambodia, Vietnam, and Malesia. *Ticanto* is therefore the only genus in the Caesalpinia Group of which most species occur either partially or exclusively in China.

## Supplementary Material

XML Treatment for Ticanto

XML Treatment for Ticanto caesia

XML Treatment for Ticanto crista

XML Treatment for Ticanto elliptifolia

XML Treatment for Ticanto magnifoliolata

XML Treatment for Ticanto rhombifolia

XML Treatment for Ticanto sinensis

XML Treatment for Ticanto szechuenensis

XML Treatment for Ticanto vernalis

XML Treatment for Ticanto yunnanensis

## Supplementary materials

Supplementary material 1 Appendix 1 Data type Occurrences. Explanation note **Table S1.** Specimens used for mapping point localities. https://binary.pensoft.net/file/732140This dataset is made available under the Open Database License (http://opendatacommons.org/licenses/odbl/1.0/). The Open Database License (ODbL) is a license agreement intended to allow users to freely share, modify, and use this Dataset while maintaining this same freedom for others, provided that the original source and author(s) are credited.Ruth P. Clark, Kai-Wen Jiang, Edeline Gagnon

Supplementary material 2 Appendix 2 Data type Docx file. Explanation note **Table S2**. Sequences obtained from GenBank. https://binary.pensoft.net/file/732141This dataset is made available under the Open Database License (http://opendatacommons.org/licenses/odbl/1.0/). The Open Database License (ODbL) is a license agreement intended to allow users to freely share, modify, and use this Dataset while maintaining this same freedom for others, provided that the original source and author(s) are credited.Ruth P. Clark

Supplementary material 3 Caesalpinia group Bayesian phylogeny Data type Phylogeny. Explanation note Caesalpinia group Bayesian phylogeny based on the combined dataset. https://binary.pensoft.net/file/732142This dataset is made available under the Open Database License (http://opendatacommons.org/licenses/odbl/1.0/). The Open Database License (ODbL) is a license agreement intended to allow users to freely share, modify, and use this Dataset while maintaining this same freedom for others, provided that the original source and author(s) are credited.Ruth P. Clark

Supplementary material 4 Caesalpinia group ML phylogeny Data type Phylogeny. Explanation note Caesalpinia group ML phylogeny based on the combined dataset, including the accession Kiyama et al. 1233, *Ticantomagnifoliolata*. https://binary.pensoft.net/file/732143This dataset is made available under the Open Database License (http://opendatacommons.org/licenses/odbl/1.0/). The Open Database License (ODbL) is a license agreement intended to allow users to freely share, modify, and use this Dataset while maintaining this same freedom for others, provided that the original source and author(s) are credited.Ruth P. Clark
