# Supplementary material for: ﻿Reinstatement of Ticanto (Leguminosae-Caesalpinioideae) – the final piece in the Caesalpinia group puzzle
Source: PhytoKeys. 2022 Aug 22;205:59–98. doi: 10.3897/phytokeys.205.82300 (PMC9849013; doi:10.3897/phytokeys.205.82300)
Supplement: Supplementary material 2 — Appendix 2 [file phytokeys-205-059_article-82300__-s002.docx]

**Table 3. Sequences obtained from GenBank**

|  |  |  |  |  |  | **Genbank number** | | | | |  |
| --- | --- | --- | --- | --- | --- | --- | --- | --- | --- | --- | --- |
|  | **Genus** | **species** | **Collector name** | **Collector number** | **Herbarium** | ***ITS*** | ***trnL-trnF*** | ***matK-trnK*** | ***rps16*** | ***trnD-trnT*** | ***ycf6-psbM*** |
|  | *Arquita* | *celendiniana* | Hughes et al. | 2210 | FHO | [KP003650](https://www.ncbi.nlm.nih.gov/nuccore/KP003650.1?report=fasta) | [KX373114](https://www.ncbi.nlm.nih.gov/nuccore/KX373114.1?report=fasta) | [KX176805](https://www.ncbi.nlm.nih.gov/nuccore/KX176805.1?report=fasta) | [KF522148](https://www.ncbi.nlm.nih.gov/nuccore/KF522148.1?report=fasta) | [KP003756](https://www.ncbi.nlm.nih.gov/nuccore/KP003756.1?report=fasta) | [KP003703](https://www.ncbi.nlm.nih.gov/nuccore/KP003703) |
|  | *Balsamocarpon* | *brevifolium* | Baxter | 1869 | E | [KP003689](https://www.ncbi.nlm.nih.gov/nuccore/KP003689) | [EU361739](https://www.ncbi.nlm.nih.gov/nuccore/EU361739) | [EU361864](https://www.ncbi.nlm.nih.gov/nuccore/EU361864) | [KF522135](https://www.ncbi.nlm.nih.gov/nuccore/KF522135) | [KP003801](https://www.ncbi.nlm.nih.gov/nuccore/KP003801) | [KP003743](https://www.ncbi.nlm.nih.gov/nuccore/KP003743) |
|  | *Biancaea* | *decapetala* | Herendeen | 19-XII- 97-1 | US | [KX372921](https://www.ncbi.nlm.nih.gov/nuccore/KX372921) | [KX373098](https://www.ncbi.nlm.nih.gov/nuccore/KX373098) | [KX176817](https://www.ncbi.nlm.nih.gov/nuccore/KX176817) | [KF522082](https://www.ncbi.nlm.nih.gov/nuccore/KF522082) | [KX379354](https://www.ncbi.nlm.nih.gov/nuccore/KX379354) | [KX372979](https://www.ncbi.nlm.nih.gov/nuccore/KX372979) |
|  | *Biancaea* | *oppositifolia* | Lugas | 921 | K | [KX372810](https://www.ncbi.nlm.nih.gov/nuccore/KX372810) | - | - | [KF522055](https://www.ncbi.nlm.nih.gov/nuccore/KF522055) | [KX379356](https://www.ncbi.nlm.nih.gov/nuccore/KX379356) | [KX372970](https://www.ncbi.nlm.nih.gov/nuccore/KX372970) |
|  | *Caesalpinia* | *anacantha* | Liogier | 16639 | P | [KX372859](https://www.ncbi.nlm.nih.gov/nuccore/KX372859) | - | - | [KX373127](https://www.ncbi.nlm.nih.gov/nuccore/KX373127) | [KX379263](https://www.ncbi.nlm.nih.gov/nuccore/KX379263) | - |
|  | *Caesalpinia* | *cassioides* | Pennington | 789 | E | [KX372858](https://www.ncbi.nlm.nih.gov/nuccore/KX372858.1?report=fasta) | [AF430711](https://www.ncbi.nlm.nih.gov/nuccore/AF430711.1?report=fasta) | - | [KF522096](https://www.ncbi.nlm.nih.gov/nuccore/KF522096.1?report=fasta) | [KX379361](https://www.ncbi.nlm.nih.gov/nuccore/KX379361.1?report=fasta) | [KX373034](https://www.ncbi.nlm.nih.gov/nuccore/KX373034) |
|  | *Caesalpinia* | *crista* | Herendeen | 1-V-99-3 | US | [KX372807](https://www.ncbi.nlm.nih.gov/nuccore/KX372807) | [KX373094](https://www.ncbi.nlm.nih.gov/nuccore/KX373094) | [EU361900](https://www.ncbi.nlm.nih.gov/nuccore/EU361900) | [KF522073](https://www.ncbi.nlm.nih.gov/nuccore/KF522073) | [KX379384](https://www.ncbi.nlm.nih.gov/nuccore/KX379384) | [KX372971](https://www.ncbi.nlm.nih.gov/nuccore/KX372971) |
|  | *Caesalpinia* | *crista* | Wieringa et al. | 4199 | WAG | [KX372808](https://www.ncbi.nlm.nih.gov/nuccore/KX372808) | - | [KX538530](https://www.ncbi.nlm.nih.gov/nuccore/KX538530.1) | [KF522074](https://www.ncbi.nlm.nih.gov/nuccore/KF522074) | [KX379385](https://www.ncbi.nlm.nih.gov/nuccore/KX379385) | [KX372972](https://www.ncbi.nlm.nih.gov/nuccore/KX372972) |
|  | *Caesalpinia* | *nipensis* | Lewis | 1838 | K | [KX372864](https://www.ncbi.nlm.nih.gov/nuccore/KX372864) | - | [KX176835](https://www.ncbi.nlm.nih.gov/nuccore/KX176835) | [KX373128](https://www.ncbi.nlm.nih.gov/nuccore/KX373128) | [KX379413](https://www.ncbi.nlm.nih.gov/nuccore/KX379413) | [KX372980](https://www.ncbi.nlm.nih.gov/nuccore/KX372980) |
|  | *Caesalpinia* | *pulcherrima* | Fougere-Danezan | 19 | MT | - | [EU361774](https://www.ncbi.nlm.nih.gov/nuccore/EU361774) | [KX176820](https://www.ncbi.nlm.nih.gov/nuccore/KX176820) | [KF522172](https://www.ncbi.nlm.nih.gov/nuccore/KF522172) | [KX379363](https://www.ncbi.nlm.nih.gov/nuccore/KX379363) | [KX373031](https://www.ncbi.nlm.nih.gov/nuccore/KX373031) |
|  | *Cassia* | *ferruginea* var. *vellozana* | Fougere-Danezan | 6 | MT | [KX372778](https://www.ncbi.nlm.nih.gov/nuccore/KX372778) | [KF794173](https://www.ncbi.nlm.nih.gov/nuccore/KF794173) | [EU361910](https://www.ncbi.nlm.nih.gov/nuccore/EU361910) | [KF522255](https://www.ncbi.nlm.nih.gov/nuccore/KF522255) | [KX379272](https://www.ncbi.nlm.nih.gov/nuccore/KX379272) | [KX372932](https://www.ncbi.nlm.nih.gov/nuccore/KX372932) |
|  | *Cenostigma* | *eriostachys* | Lewis | 1799 | K | [KX372836](https://www.ncbi.nlm.nih.gov/nuccore/KX372836) | - | - | [KF522029](https://www.ncbi.nlm.nih.gov/nuccore/KF522029) | [KX379444](https://www.ncbi.nlm.nih.gov/nuccore/KX379444) | [KX372993](https://www.ncbi.nlm.nih.gov/nuccore/KX372993) |
|  | *Cenostigma* | *tocantinum* | Klitgaard & Lewis | 88 | K | [KP003694](https://www.ncbi.nlm.nih.gov/nuccore/KP003694) | - | [KX176806](https://www.ncbi.nlm.nih.gov/nuccore/KX176806) | [KF522071](https://www.ncbi.nlm.nih.gov/nuccore/KF522071) | [KP003803](https://www.ncbi.nlm.nih.gov/nuccore/KP003803) | [KP003740](https://www.ncbi.nlm.nih.gov/nuccore/KP003740) |
|  | *Cordeauxia* | *edulis* | Kuchar | 17803 | K | [KX372826](https://www.ncbi.nlm.nih.gov/nuccore/KX372826) | [EU361787](https://www.ncbi.nlm.nih.gov/nuccore/EU361787) | [EU361920](https://www.ncbi.nlm.nih.gov/nuccore/EU361920) | [KF522084](https://www.ncbi.nlm.nih.gov/nuccore/KF522084) | [KX379430](https://www.ncbi.nlm.nih.gov/nuccore/KX379430) | [KX372998](https://www.ncbi.nlm.nih.gov/nuccore/KX372998) |
|  | *Coulteria* | *mollis* | Way | 28 | K | [KX372887](https://www.ncbi.nlm.nih.gov/nuccore/KX372887) | - | - | [KF522187](https://www.ncbi.nlm.nih.gov/nuccore/KF522187) | [KX379403](https://www.ncbi.nlm.nih.gov/nuccore/KX379403) | [KX373051](https://www.ncbi.nlm.nih.gov/nuccore/1091543909) |
|  | *Coulteria* | *platyloba* | Gagnon & Marazzi | EG2010.007 | MT | [KX372894](https://www.ncbi.nlm.nih.gov/nuccore/KX372894) | - | [KM219821](https://www.ncbi.nlm.nih.gov/nuccore/KM219821) | [KF522175](https://www.ncbi.nlm.nih.gov/nuccore/KF522175) | [KX379407](https://www.ncbi.nlm.nih.gov/nuccore/KX379407) | [KX373057](https://www.ncbi.nlm.nih.gov/nuccore/KX373057) |
|  | *Coulteria* | *pumila* | Lewis | 2067 | K | [KF379234](https://www.ncbi.nlm.nih.gov/nuccore/KF379234) |  | [KX176832](https://www.ncbi.nlm.nih.gov/nuccore/KX176832) | [KF522177](https://www.ncbi.nlm.nih.gov/nuccore/KF522177) | [KX379406](https://www.ncbi.nlm.nih.gov/nuccore/KX379406) | [KX373055](https://www.ncbi.nlm.nih.gov/nuccore/KX373055) |
|  | *Denisophytum* | *eriantherum* | Friis et al. | 4698 | K | [KX372878](https://www.ncbi.nlm.nih.gov/nuccore/KX372878) | - | - | [KF522123](https://www.ncbi.nlm.nih.gov/nuccore/KF522123) | [KX379333](https://www.ncbi.nlm.nih.gov/nuccore/KX379333) | [KX373023](https://www.ncbi.nlm.nih.gov/nuccore/KX373023) |
|  | *Denisophytum* | *stuckertii* | Beck | 9443 | NY | [KX372869](https://www.ncbi.nlm.nih.gov/nuccore/KX372869) | [KX373095](https://www.ncbi.nlm.nih.gov/nuccore/KX373095) | - | [KF522126](https://www.ncbi.nlm.nih.gov/nuccore/KF522126) | [KX379337](https://www.ncbi.nlm.nih.gov/nuccore/KX379337) | [KX373019](https://www.ncbi.nlm.nih.gov/nuccore/KX373019) |
|  | *Erythrostemon* | *gilliesii* | Spellenberg | 12701 | MT | [KP003681](https://www.ncbi.nlm.nih.gov/nuccore/KP003681) | [JX073265](https://www.ncbi.nlm.nih.gov/nuccore/JX073265) | [JX099328](https://www.ncbi.nlm.nih.gov/nuccore/JX099328) | [KF522296](https://www.ncbi.nlm.nih.gov/nuccore/KF522296) | [KP003786](https://www.ncbi.nlm.nih.gov/nuccore/KP003786) | [KP003729](https://www.ncbi.nlm.nih.gov/nuccore/KP003729) |
|  | ***Erythrostemon*** | ***mexicanus*** | **Hughes** | **1606** | **NY** | **-** | [**EU361772**](https://www.ncbi.nlm.nih.gov/nuccore/EU361772) | [**EU361904**](https://www.ncbi.nlm.nih.gov/nuccore/EU361904) | [**KF522218**](https://www.ncbi.nlm.nih.gov/nuccore/KF522218) | [**KX379296**](https://www.ncbi.nlm.nih.gov/nuccore/KX379296) | [**KX373061**](https://www.ncbi.nlm.nih.gov/nuccore/KX373061) |
|  | ***Erythrostemon*** | ***mexicanus*** | **Lewis** | **s.n. 1973-21714** | **K** | [**KP003683**](https://www.ncbi.nlm.nih.gov/nuccore/KP003683) | **-** | **-** | **-** | **-** | [**KP003730**](https://www.ncbi.nlm.nih.gov/nuccore/KP003730) |
|  | *Erythrostemon* | *palmeri* | Lewis | 2065 | K | [KP003685](https://www.ncbi.nlm.nih.gov/nuccore/KP003685) | [KX373113](https://www.ncbi.nlm.nih.gov/nuccore/KX373113) | [KF379243](https://www.ncbi.nlm.nih.gov/nuccore/KF379243) | [KF522231](https://www.ncbi.nlm.nih.gov/nuccore/KF522231) | [KP003790](https://www.ncbi.nlm.nih.gov/nuccore/KP003790) | [KP003732](https://www.ncbi.nlm.nih.gov/nuccore/KP003732) |
|  | *Gelrebia* | *bracteata* | van Hoepen | 2018 | K | [KX372784](https://www.ncbi.nlm.nih.gov/nuccore/KX372784) | - | - | [KF522258](https://www.ncbi.nlm.nih.gov/nuccore/KF522258) | [KX379345](https://www.ncbi.nlm.nih.gov/nuccore/KX379345) | [KX372952](https://www.ncbi.nlm.nih.gov/nuccore/KX372952) |
|  | *Gelrebia* | *trothaei* subsp. *erlangeri* | Beckett & White | 1711 | K | [KX372789](https://www.ncbi.nlm.nih.gov/nuccore/KX372789) | - | [KX176812](https://www.ncbi.nlm.nih.gov/nuccore/KX176812) | [KF522263](https://www.ncbi.nlm.nih.gov/nuccore/KF522263) | [KX379349](https://www.ncbi.nlm.nih.gov/nuccore/KX379349) | [KX372948](https://www.ncbi.nlm.nih.gov/nuccore/KX372948) |
|  | *Guilandina* | *bonduc* | Bruneau | 1342 | MT | [KX372797](https://www.ncbi.nlm.nih.gov/nuccore/KX372797) | - | [KX176816](https://www.ncbi.nlm.nih.gov/nuccore/KX176816) | [KF522062](https://www.ncbi.nlm.nih.gov/nuccore/KF522062) | [KX379370](https://www.ncbi.nlm.nih.gov/nuccore/KX379370) | [KX372967](https://www.ncbi.nlm.nih.gov/nuccore/KX372967) |
|  | *Guilandina* | *minax* | Li Shi Jin | 802 | IBSC | [KX372926](https://www.ncbi.nlm.nih.gov/nuccore/KX372926) | - | - | [KF522131](https://www.ncbi.nlm.nih.gov/nuccore/KF522131) | [KX379369](https://www.ncbi.nlm.nih.gov/nuccore/KX379369) | - |
|  | ***Haematoxylum*** | ***brasiletto*** | **Gagnon & Marazzi** | **EG2010.013** | **MT** | [**KX372833**](https://www.ncbi.nlm.nih.gov/nuccore/KX372833) | **-** | **-** | [**KF522206**](https://www.ncbi.nlm.nih.gov/nuccore/KF522206) | [**KX379326**](https://www.ncbi.nlm.nih.gov/nuccore/KX379326) | [**KX373040**](https://www.ncbi.nlm.nih.gov/nuccore/KX373040) |
|  | ***Haematoxylum*** | ***brasiletto*** | **Haston** | **V200307 (RNG), OFI 14/83 (OFI)** | **RNG, OFI** | **-** | [**AY899696**](https://www.ncbi.nlm.nih.gov/nuccore/AY899696) | **-** | **-** | **-** | **-** |
|  | ***Haematoxylum*** | ***brasiletto*** | **Wojciechowski** | **953** | **ASU** | **-** | **-** | [**AY386905**](https://www.ncbi.nlm.nih.gov/nuccore/AY386905) | **-** | **-** | **-** |
|  | *Haematoxylum* | *campechianum* | Miller & Morello | 8849 | MO | [KX372832](https://www.ncbi.nlm.nih.gov/nuccore/KX372832) | - | - | [KF522201](https://www.ncbi.nlm.nih.gov/nuccore/KF522201) | [KX379328](https://www.ncbi.nlm.nih.gov/nuccore/KX379328) | [KX373038](https://www.ncbi.nlm.nih.gov/nuccore/KX373038) |
|  | *Hoffmannseggia* | *glauca* | Spellenberg | 12699 | MT | [KP003690](https://rbgkew-my.sharepoint.com/personal/r_clark_kew_org/Documents/Mezoneuron/Mezo%20subgen%20nov/Revised%20manuscript/Mezoneuron%20subgen%20nov_2-1.docx) | [AF365069](https://www.ncbi.nlm.nih.gov/nuccore/AF365069) | [EU361969](https://www.ncbi.nlm.nih.gov/nuccore/EU361969) | [KF522213](https://www.ncbi.nlm.nih.gov/nuccore/KF522213) | [KP003796](https://www.ncbi.nlm.nih.gov/nuccore/KP003796) | [KP003744](https://www.ncbi.nlm.nih.gov/nuccore/KP003744) |
|  | *Hoffmannseggia* | *prostrata* | Hughes & Daza | 2359 | FHO | [KX372794](https://www.ncbi.nlm.nih.gov/nuccore/KX372794) | - | - | [KF522241](https://www.ncbi.nlm.nih.gov/nuccore/KF522241) | [KX379323](https://www.ncbi.nlm.nih.gov/nuccore/KX379323) | [KX372944](https://www.ncbi.nlm.nih.gov/nuccore/KX372944) |
|  | ***Hoffmannseggia*** | ***viscosa*** | **Hughes** | **2221** | **FHO** | [**KX372925**](https://www.ncbi.nlm.nih.gov/nuccore/KX372925) | **-** | **-** | [**KF522137**](https://www.ncbi.nlm.nih.gov/nuccore/KF522137) | [**KX379316**](https://www.ncbi.nlm.nih.gov/nuccore/KX379316) | [**KX372939**](https://www.ncbi.nlm.nih.gov/nuccore/KX372939) |
|  | ***Hoffmannseggia*** | ***viscosa*** | **Richardson** | **2039** | **NY** | [**AY308578**](https://www.ncbi.nlm.nih.gov/nuccore/AY308578) | [**AY308499**](https://www.ncbi.nlm.nih.gov/nuccore/AY308499) | **-** | **-** | **-** | **-** |
|  | *Hultholia* | *mimosoides* | R. Clark | 237 | K | - | [KX373093](https://www.ncbi.nlm.nih.gov/nuccore/KX373093) | - | [KX373148](https://www.ncbi.nlm.nih.gov/nuccore/KX373148) | [KX379262](https://www.ncbi.nlm.nih.gov/nuccore/KX379262) | [KX372954](https://www.ncbi.nlm.nih.gov/nuccore/KX372954) |
|  | *Libidibia* | *monosperma* | Gardner | 7029 | E | - | [EU361838](https://www.ncbi.nlm.nih.gov/nuccore/EU361838) | [EU362050](https://www.ncbi.nlm.nih.gov/nuccore/EU362050) | [KX373142](https://www.ncbi.nlm.nih.gov/nuccore/KX373142) | [KX379422](https://www.ncbi.nlm.nih.gov/nuccore/KX379422) | [KX373009](https://www.ncbi.nlm.nih.gov/nuccore/KX373009) |
|  | *Libidibia* | *paraguariensis* | Lewis & Klitgaard | 2170 | K | [KF379233](https://www.ncbi.nlm.nih.gov/nuccore/KF379233) | [KX373119](https://www.ncbi.nlm.nih.gov/nuccore/KX373119) | [EU361905](https://www.ncbi.nlm.nih.gov/nuccore/EU361905) | [KF522112](https://www.ncbi.nlm.nih.gov/nuccore/KF522112) | [KX379419](https://www.ncbi.nlm.nih.gov/nuccore/KX379419) | [KX373005](https://www.ncbi.nlm.nih.gov/nuccore/KX373005) |
|  | *Lophocarpinia* | *aculeatifolia* | Fortunato | 8639 | BAB | - | [JX219460](https://www.ncbi.nlm.nih.gov/nuccore/JX219460) | [JX219466](https://www.ncbi.nlm.nih.gov/nuccore/JX219466) | - | - | - |
|  | *Mezoneuron* | *cucullatum* | Grierson & Long | 3623 | K | [KX372819](https://www.ncbi.nlm.nih.gov/nuccore/KX372819) | - | - | [KF522194](https://www.ncbi.nlm.nih.gov/nuccore/KF522194) | [KX379266](https://www.ncbi.nlm.nih.gov/nuccore/KX379266) | - |
|  | *Mezoneuron* | *hildebrandtii* | Lewis et al. | 2137 | K | [KX372816](https://www.ncbi.nlm.nih.gov/nuccore/KX372816) | [KX373107](https://www.ncbi.nlm.nih.gov/nuccore/KX373107) | [KU245661](https://www.ncbi.nlm.nih.gov/nuccore/KU245661) | [KF522198](https://www.ncbi.nlm.nih.gov/nuccore/KF522198) | [KX379386](https://www.ncbi.nlm.nih.gov/nuccore/KX379386) | [KX372958](https://www.ncbi.nlm.nih.gov/nuccore/KX372958) |
|  | *Mezoneuron* | *kauaiense* | Lorence & Wagner | 8904 | NTBG | [KX372823](https://www.ncbi.nlm.nih.gov/nuccore/KX372823) | - | - | [KF522192](https://www.ncbi.nlm.nih.gov/nuccore/KF522192) | [KX379391](https://www.ncbi.nlm.nih.gov/nuccore/KX379391) | [KX372961](https://www.ncbi.nlm.nih.gov/nuccore/KX372961) |
|  | *Moullava* | *digyna* | Maxwell | 91-827 | P | [KX372803](https://www.ncbi.nlm.nih.gov/nuccore/KX372803) | - | - | [KX373146](https://www.ncbi.nlm.nih.gov/nuccore/KX373146) | [KX379383](https://www.ncbi.nlm.nih.gov/nuccore/KX379383) | - |
|  | *Moullava* | *spicata* | Critchett | 11/79 | K | [KX372805](https://www.ncbi.nlm.nih.gov/nuccore/KX372805) | [JX073267](https://www.ncbi.nlm.nih.gov/nuccore/JX073267) | [KX176818](https://www.ncbi.nlm.nih.gov/nuccore/KX176818) | [KF522252](https://www.ncbi.nlm.nih.gov/nuccore/KF522252) | [KX379378](https://www.ncbi.nlm.nih.gov/nuccore/KX379378) | - |
|  | *Pomaria* | *jamesii* | Higgins | 17628 | NY | [KP003677](https://www.ncbi.nlm.nih.gov/nuccore/KP003677) | [EU361830](https://www.ncbi.nlm.nih.gov/nuccore/EU361830) | [EU362029](https://www.ncbi.nlm.nih.gov/nuccore/EU362029) | [KF522090](https://www.ncbi.nlm.nih.gov/nuccore/KF522090) | [KP003793](https://www.ncbi.nlm.nih.gov/nuccore/KP003793) | [KP003736](https://www.ncbi.nlm.nih.gov/nuccore/KP003736) |
|  | ***Pterogyne*** | ***nitens*** | **Herendeen** | **13-XII-97-1** | **US** | [**KX372782**](https://www.ncbi.nlm.nih.gov/nuccore/KX372782) | **-** | [**EU362031**](https://www.ncbi.nlm.nih.gov/nuccore/EU362031) | **-** | [**KX379276**](https://www.ncbi.nlm.nih.gov/nuccore/KX379276) | [**KX372936**](https://www.ncbi.nlm.nih.gov/nuccore/KX372936) |
|  | ***Pterogyne*** | ***nitens*** | **Pennington** | **244** | **FHO** | **-** | [**AY899689**](https://www.ncbi.nlm.nih.gov/nuccore/AY899689) | **-** | [**AY899747**](https://www.ncbi.nlm.nih.gov/nuccore/AY899747) | **-** | **-** |
|  | *Pterolobium* | *hexapetalum* | Grierson & Long | 2075 | P | [KX372806](https://www.ncbi.nlm.nih.gov/nuccore/KX372806) | - | - | [KX373139](https://www.ncbi.nlm.nih.gov/nuccore/KX373139) | - | [KX372973](https://www.ncbi.nlm.nih.gov/nuccore/KX372973) |
|  | *Pterolobium* | *integrum* | van Beusekom | 4021 | P | - | - | - | - | [KX379456](https://www.ncbi.nlm.nih.gov/nuccore/KX379456) | - |
|  | *Pterolobium* | *macropterum* | Grierson & Long | 1624 | P | - | - | - | [KX373141](https://www.ncbi.nlm.nih.gov/nuccore/KX373141) | [KX379454](https://www.ncbi.nlm.nih.gov/nuccore/KX379454) | [KX372974](https://www.ncbi.nlm.nih.gov/nuccore/KX372974) |
|  | *Pterolobium* | *macropterum* | Geesink & al. | 5934 | P | - | - | - | [KX373140](https://www.ncbi.nlm.nih.gov/nuccore/KX373140) | [KX379455](https://www.ncbi.nlm.nih.gov/nuccore/KX379455) | - |
|  | *Pterolobium* | *stellatum* | Herendeen | 17-XII-97-9 | US | [KX372812](https://www.ncbi.nlm.nih.gov/nuccore/KX372812) | [KX373115](https://www.ncbi.nlm.nih.gov/nuccore/KX373115) | [EU362032](https://www.ncbi.nlm.nih.gov/nuccore/EU362032) | [KF522238](https://www.ncbi.nlm.nih.gov/nuccore/KF522238) | [KX379457](https://www.ncbi.nlm.nih.gov/nuccore/KX379457) | - |
|  | *Stenodrepanum* | *bergii* | Hick & Bertone | 8 | CORD | ***-*** | [**AF430788**](https://www.ncbi.nlm.nih.gov/nuccore/AF430788) | [**JX219467**](https://www.ncbi.nlm.nih.gov/nuccore/JX219467) | ***-*** | ***-*** | ***-*** |
|  | *Stuhlmannia* | *moavi* | Luke | 3710 | MO, K | [**KX372829**](https://www.ncbi.nlm.nih.gov/nuccore/KX372829) | **-** | **-** | [**KF522061**](https://www.ncbi.nlm.nih.gov/nuccore/KF522061) | [**KX379431**](https://www.ncbi.nlm.nih.gov/nuccore/KX379431) | [**KX373001**](https://www.ncbi.nlm.nih.gov/nuccore/KX373001) |
|  | ***Tara*** | ***cacalaco*** | **Lewis** | **1789** | **K** | **-** | [**AF365063**](https://www.ncbi.nlm.nih.gov/nuccore/AF365063) | [**EU361898**](https://www.ncbi.nlm.nih.gov/nuccore/EU361898) | **-** | **-** | **-** |
|  | ***Tara*** | ***cacalaco*** | **Walker** | **s.n. 1986-6481** | **K** | [**KX372886**](https://www.ncbi.nlm.nih.gov/nuccore/KX372886) | **-** | **-** | **-** | [**KX379397**](https://www.ncbi.nlm.nih.gov/nuccore/KX379397) | [**KX373048**](https://www.ncbi.nlm.nih.gov/nuccore/KX373048) |
|  | ***Tara*** | ***spinosa*** | **Eastwood** | **36** | **FHO** | **-** | **-** | [**KF379250**](https://www.ncbi.nlm.nih.gov/nuccore/KF379250) | **-** | **-** | [**KX373046**](https://www.ncbi.nlm.nih.gov/nuccore/KX373046) |
|  | ***Tara*** | ***spinosa*** | **Hughes et al.** | **2360** | **FHO** | [**KX372881**](https://www.ncbi.nlm.nih.gov/nuccore/KX372881) | **-** | **-** | [**KF522129**](https://www.ncbi.nlm.nih.gov/nuccore/KF522129) | [**KX379399**](https://www.ncbi.nlm.nih.gov/nuccore/KX379399) | [**KX373045**](https://www.ncbi.nlm.nih.gov/nuccore/KX373045) |
|  | *Tara* | *vesicaria* | Hawkins & Hughes | 11 | FHO | [**KX372882**](https://www.ncbi.nlm.nih.gov/nuccore/KX372882) | **-** | **-** | **-** | [**KX379395**](https://www.ncbi.nlm.nih.gov/nuccore/KX379395) | [**KX373049**](https://www.ncbi.nlm.nih.gov/nuccore/KX373049) |
|  | *Zuccagnia* | *punctata* | Galletto | 171 | CORD | [**KP003688**](https://www.ncbi.nlm.nih.gov/nuccore/KP003688) | **-** | [**KX176813**](https://www.ncbi.nlm.nih.gov/nuccore/KX176813) | [**KF522141**](https://www.ncbi.nlm.nih.gov/nuccore/KF522141) | [**KP003798**](https://www.ncbi.nlm.nih.gov/nuccore/KP003798) | [**KP003742**](https://www.ncbi.nlm.nih.gov/nuccore/KP003742) |
