# Supplementary figures and images for: ﻿Reinstatement of Ticanto (Leguminosae-Caesalpinioideae) – the final piece in the Caesalpinia group puzzle
Source: PhytoKeys. 2022 Aug 22;205:59–98. doi: 10.3897/phytokeys.205.82300 (PMC9849013; doi:10.3897/phytokeys.205.82300)

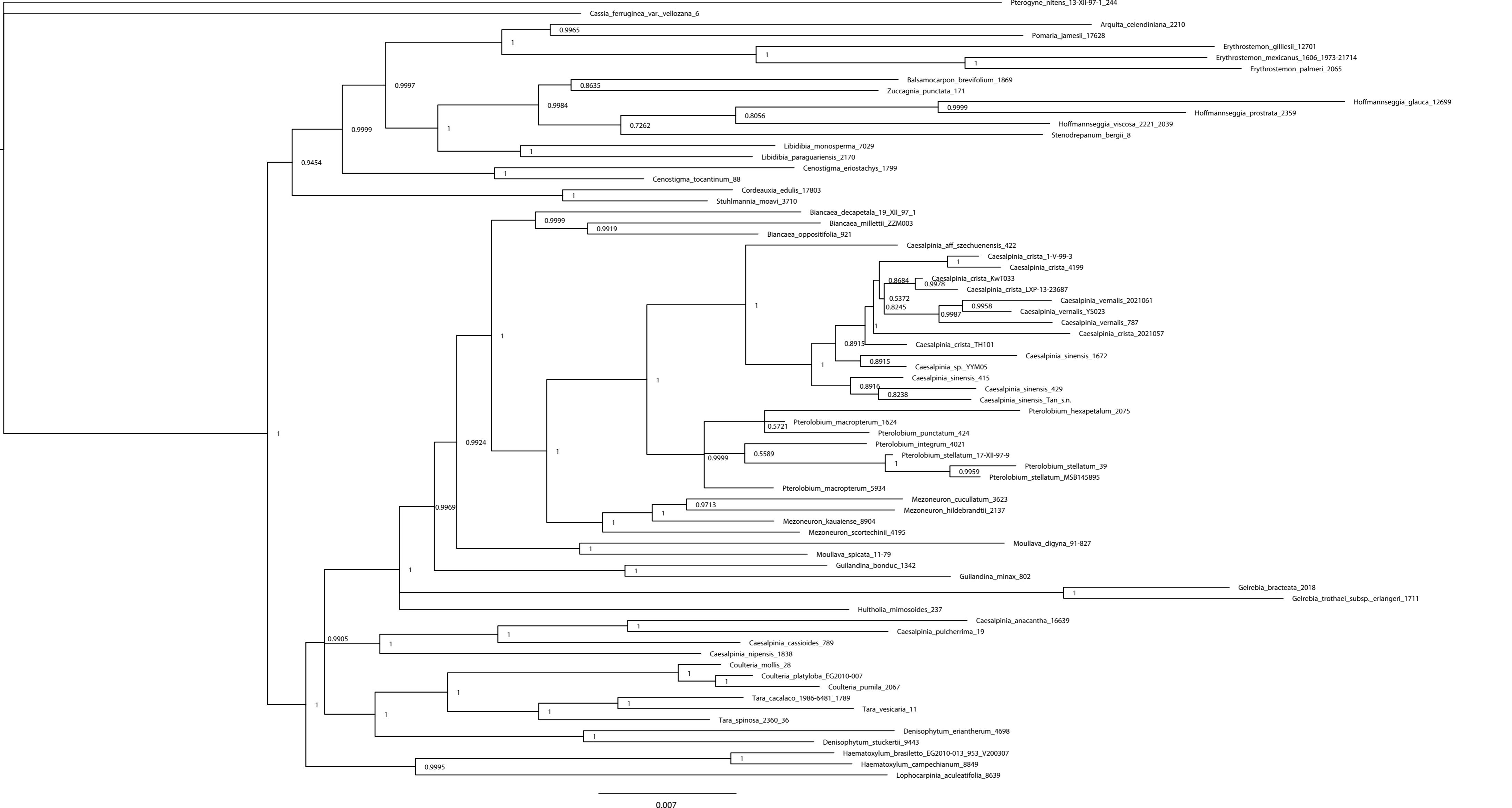

Supplement: Supplementary material 3 — Caesalpinia group Bayesian phylogeny [file phytokeys-205-059_article-82300__-s003.pdf]

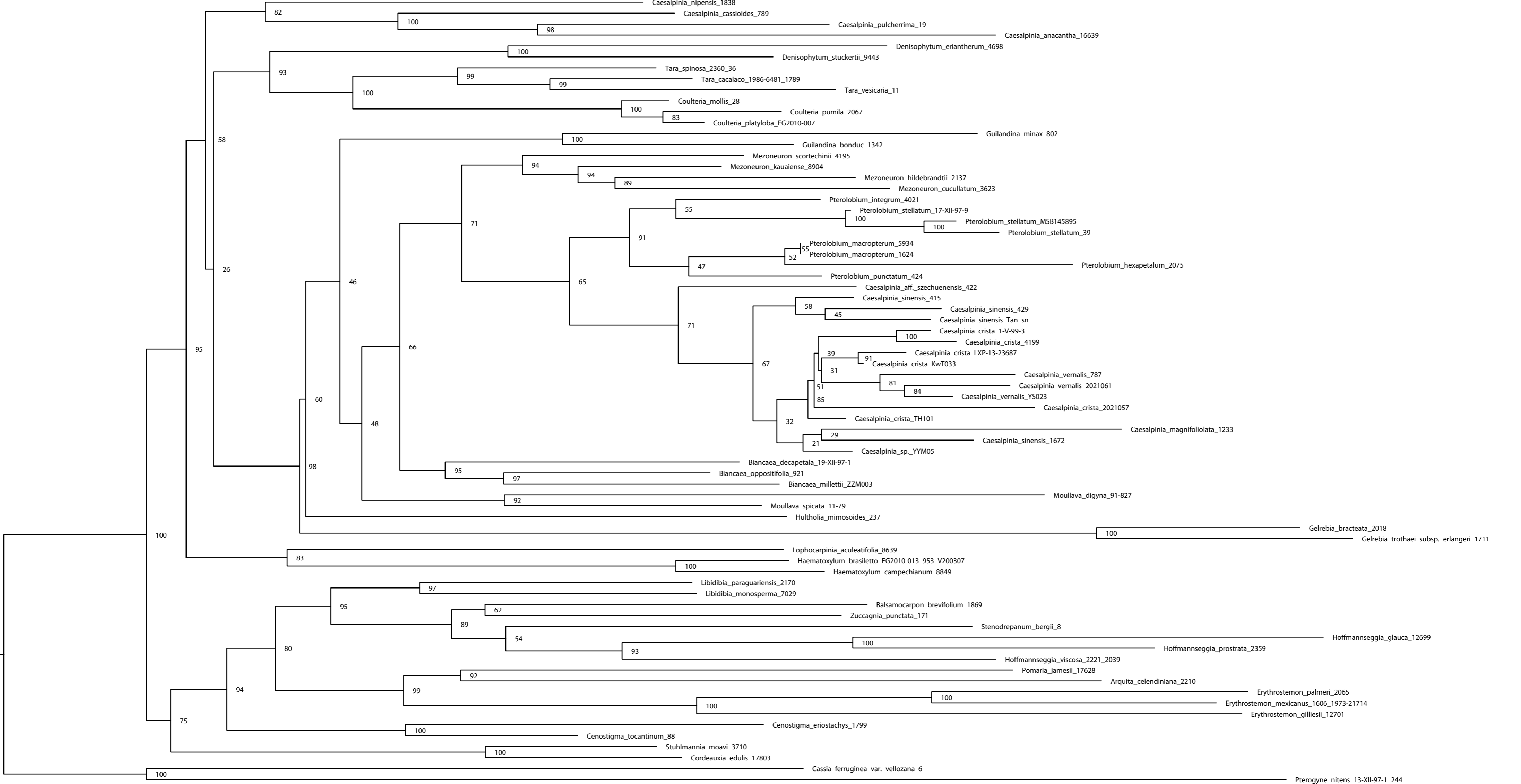

0.02

Supplement: Supplementary material 4 — Caesalpinia group ML phylogeny [file phytokeys-205-059_article-82300__-s004.pdf]
